# Molecular Mapping of Movement-Associated Areas in the Avian Brain: A Motor Theory for Vocal Learning Origin

**DOI:** 10.1371/journal.pone.0001768

**Published:** 2008-03-12

**Authors:** Gesa Feenders, Miriam Liedvogel, Miriam Rivas, Manuela Zapka, Haruhito Horita, Erina Hara, Kazuhiro Wada, Henrik Mouritsen, Erich D. Jarvis

**Affiliations:** 1 Volkswagen Nachwuchsgruppe Animal Navigation, Institut für Biologie und Umweltwissenschaften (IBU), University of Oldenburg, Oldenburg, Germany; 2 Duke University Medical Center, Department of Neurobiology, Durham, North Carolina, United States of America; University of Cambridge, United Kingdom

## Abstract

Vocal learning is a critical behavioral substrate for spoken human language. It is a rare trait found in three distantly related groups of birds-songbirds, hummingbirds, and parrots. These avian groups have remarkably similar systems of cerebral vocal nuclei for the control of learned vocalizations that are not found in their more closely related vocal non-learning relatives. These findings led to the hypothesis that brain pathways for vocal learning in different groups evolved independently from a common ancestor but under pre-existing constraints. Here, we suggest one constraint, a pre-existing system for movement control. Using behavioral molecular mapping, we discovered that in songbirds, parrots, and hummingbirds, all cerebral vocal learning nuclei are adjacent to discrete brain areas active during limb and body movements. Similar to the relationships between vocal nuclei activation and singing, activation in the adjacent areas correlated with the amount of movement performed and was independent of auditory and visual input. These same movement-associated brain areas were also present in female songbirds that do not learn vocalizations and have atrophied cerebral vocal nuclei, and in ring doves that are vocal non-learners and do not have cerebral vocal nuclei. A compilation of previous neural tracing experiments in songbirds suggests that the movement-associated areas are connected in a network that is in parallel with the adjacent vocal learning system. This study is the first global mapping that we are aware for movement-associated areas of the avian cerebrum and it indicates that brain systems that control vocal learning in distantly related birds are directly adjacent to brain systems involved in movement control. Based upon these findings, we propose a motor theory for the origin of vocal learning, this being that the brain areas specialized for vocal learning in vocal learners evolved as a specialization of a pre-existing motor pathway that controls movement.

## Introduction

**Figure 1:**
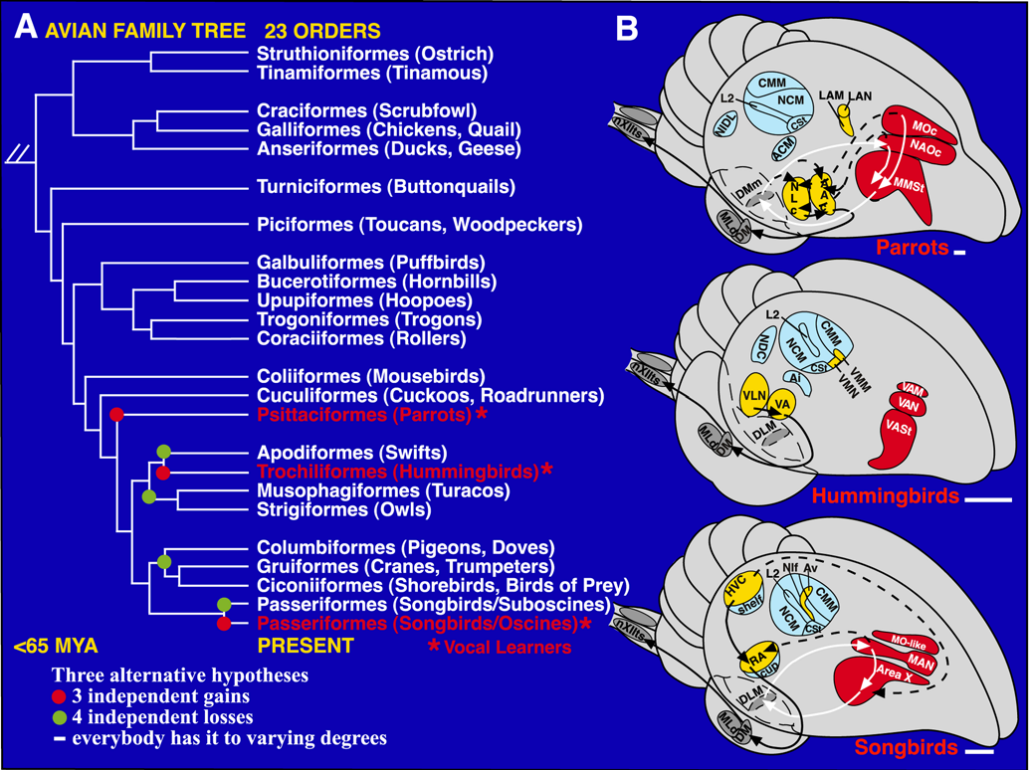
Phylogenetic and brain relationships of avian vocal learners. A. One view of the phylogenetic relationships of living birds [129]. Vocal learners are highlighted in red. Red dots: possible independent gains of vocal learning; green dots: alternatively, possible independent losses. B. Semi-3D view of seven cerebral vocal nuclei (red and yellow) found in vocal learners and of auditory areas (blue) found in all birds. Red-labeled vocal nuclei and white arrows: anterior vocal pathway. Yellow-labeled vocal nuclei and black arrows: posterior vocal pathway. Only a few connections in hummingbirds are known and that of songbird MO is not known. Based on serial sections of singing-driven IEG expression in this study, we see that NIf and Av are adjacent at lateral levels. Anterior is right, dorsal is up. Scale bars, 1 mm. Figure modified from Jarvis et al. (2000) [7] and Jarvis (2004) [10], with connectivity reviewed therein.

Vocal learning is the ability to imitate vocalization from others, and is a critical behavioral substrate for spoken human language. As such, vocal learning is a rare trait found to date in at least three distantly related groups of mammals (humans, bats, and cetaceans [1]; also see recent studies including seals [1], [2] and elephants [3]), and in three distantly related groups of birds (songbirds, hummingbirds, and parrots [4]; **Fig. 1A**). Among the birds, vocalizing-driven immediate early gene (IEG) expression experiments revealed that each vocal learning group possesses seven comparable cerebral (i.e. telencephalic) song nuclei, also called vocal nuclei (**Fig. 1B**) [5]–[7]. Three of the nuclei make up part of an anterior vocal pathway (**Fig. 1B**, red) that in songbirds is necessary for vocal learning [8]–[10]. The other four nuclei form a posterior vocal pathway (**Fig. 1B**, yellow) of which the songbird HVC and RA are necessary for the production of the learned vocalizations [10]–[12] (abbreviations in **Table 1**). None of these cerebral vocal nuclei have been found to date in vocal non-learners, such as in the suboscine songbirds closely related to songbirds [13], [14], the interrelated doves [15], [16], and the distantly related galliforms ([17], **Fig. 1B**). Yet, both vocal learners and non-learners have brainstem vocal nuclei DM and nXIIts (**Fig. 1B**) that are responsible for production of innate vocalizations [7], [18], and an auditory cerebral pathway that is responsible for processing species-specific vocalizations and auditory learning ([19]–[21], **Fig. 1B**, blue). Therefore, according to the dominant hypothesis [4], [14], within the past 65 million years 3 out of 23 avian orders independently evolved seven similar cerebral vocal nuclei for a complex behavior ([7], **Fig. 1A**, red dots). The reason for these remarkable similarities had remained mysterious, but they suggest that the evolution of brain structures for vocal learning is under strong genetic or epigenetic constraints. Here we conducted a series of experiments that, in addition to identifying the motor control circuit of the avian brain, points towards a possible genetic constraint: a pre-existing motor system that in birds consists of at least seven cerebral areas active during the production of limb and body movements. Of the multiple hypotheses proposed for the evolution of vocal learning [14], [22], [23], including for spoken language, our findings support those that suggest a motor origin [10], [24], [25].

**Table 1:** Terminology of comparable regions across avian species studied

Modality	Vocal			Movement				Visual	Auditory
Species>	Song	Parrot	Humb	Song	Parrot	Humb	Dove	All	All
**Subdivision**									
**H**	-	-	-	AH	AH	AH	AH	PH	-
**MD**	-	-	-	AMD	AMD	AMD	AMD	PMD	-
**MV**	MO	MO	VAM	AMV	AMV	AMV	AMV		
	Av	LAM	VMM	PLMV	LMV	n.d.	PLMV		
				MVb	MVb	n.d.	MVb	MVe	MV-L2
**N**	MAN	NAO	VAN	AN	AN	AN	AN		
	HVC	NLC	VLN	DLN	SLN	DLN	DLN		
	NIf	LAN	VMN	PLN	LN	n.d.	PLN		NDC
				Nb	Nb	n.d.	Nb	Ne	N-L2
**A**	RA	AAC	VA	LAI	LAI	LAI	LAI		AI
**St**	Area X	MMSt	VASt	ASt	ASt	ASt	ASt	LSte	CSt

n.d., not done. Humb, hummingbird. Song, songbird. Abbreviations are listed below.

**Abbreviations**

A, arcopallium

AAC, central nucleus of the anterior arcopallium

ACM, caudal medial arcopallium

AH, anterior hyperpallium

AI, intermediate arcopallium

AIVM, ventral medial intermediate arcopallium

AMD, anterior dorsal mesopallium

AMV, anterior ventral mesopallium

AN, anterior nidopallium

Area X, a vocal nucleus

ASt, anterior striatum

Av, nucleus avalanche

B, basorostralis

Cb, cerebellum

CM, caudal mesopallium

CSt, caudal striatum

DLN, dorsal lateral nidopallium

DLM, dorsal lateral nucleus of the thalamus

DM, dorsal medial nucleus of the midbrain

DMm, magnocellular nucleus of the dorsal thalamus

DT, dorsal thamalus

E, entopallium

GP, globus pallidus

H, hyperpallium

Hp, hippocampus

HVC, a vocal nucleus (no abbreviation)

LAI, lateral intermediate arcopallium

LAN, lateral nucleus of the anterior nidopallium

LAM, lateral nucleus of the anterior mesopallium

LMV, lateral ventral mesopallium

M, mesopallium

MAN, magnocellular nucleus of the anterior nidopallium

MLd, dorsal part of the lateral mesencephalic nucleus

MMSt, magnocellular nucleus of the medial striatum

MO, oval nucleus of the mesopallium

MD, dorsal mesopallium

MN, motor neurons

MV, ventral mesopallium

MVb, ventral mesopallium adjacent to B

MVe, ventral mesopallium adjacent to E

MV-L2, ventral mesopallium adjacent to L2 (same as CM)

N, nidopallium

Nb, nidopallium adjacent to B

ND, dorsal nidopallium

Ne, nidopallium adjacent to E

N-L2, nidopallium adjacent to L2

NAO, oval nucleus of the anterior nidopallium

NDC, caudal dorsal nidopallium

NIDL, dorsal lateral intermediate nidopallium

NIf, interfacial nucleus of the nidopallium

NLC, central nucleus of the lateral nidopallium

nXIIts, 12^th^ nucleus tracheosyringeal part

OT, optic tectum

PDN, posterior dorsal nidopallium

PH, posterior hyperpallium

PLMV, posterior lateral ventral mesopallium

PLN, posterior lateral nidopallium

PLSt, posterior lateral striatum

PDN, posterior dorsal nidopallium

PMN, pre-motor neurons

RA, robust nucleus of the arcopallium

S, septum

SLN, supra lateral nidopallium

St, striatum

Ste, striatum adjacent to E

v, ventricle

VA, vocal nucleus of the arcopallium

VAM, vocal nucleus of the anterior mesopallium

VAN, vocal nucleus of the anterior nidopallium

VASt, vocal nucleus of the anterior striatum

VMM, vocal nucleus of the medial mesopallium

VLN, vocal nucleus of the lateral nidopallium

## Results

**Figure 2:**
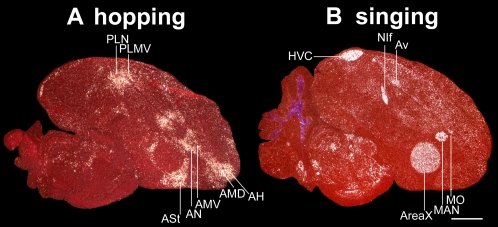
IEG expression patterns in brain sections from moving versus singing zebra finches. A. Example of the most restricted movement-associated expression pattern obtained in this study. A mid-sagittal section from a deafened bird that was hopping in the dark. B. Comparable section from a bird that was singing while movingly relatively little. ZENK expression is shown in (A); c-fos is shown in (B) due to its high contrast in vocal nuclei. White: IEG expression; red: cresyl violet staining. Six (HVC, NIf, Av, Area X, MAN, and MO) of the seven vocal nuclei can be seen in these sections, which are adjacent to five movement-associated areas (PLN, PLMV, ASt, AN, AMV); movement-associated areas laterally adjacent to the HVC and RA are not in this section. AMD and AH are known somatosensory areas. Anterior is right, dorsal is up. Scale bar, 2 mm.

In experiments that identified night vision brain areas in migratory songbirds [26], [27], we performed a series of control experiments that led to the identification of brain areas associated with movement behavior that we report here. Unexpectedly, the areas of robust movement-associated activation were closest to the vocal nuclei, and thus we investigated this activation further in a non-migratory songbird, the zebra finch (*Taeniopygia guttata*), for which the vocal system has been studied in detail. Once we established these areas as movement-associated and adjacent to vocal nuclei in songbirds (Part I of this report), we next tested whether other vocal learning birds (Part II) and vocal non-learning birds (Part III) had similar properties to address implications on the evolution of vocal learning. To perform the experiments, we compared groups of birds that repeatedly performed specific movements for 30–60 min with control groups that either sat still but awake or were in other experimental conditions. The movement behaviors were wing whirring, flights, hopping, or walking. We sectioned the brains and performed in-situ hybridizations for the activity-dependent IEG transcription factors ZENK and c-fos. The mRNA expression of these genes is sensitive to increased activity in neurons [28]. We processed adjacent sections for expression of the AMPA glutamate receptor subunit GluR1 or the transcription factor FoxP1, because we found that these anatomical markers were critical for identifying the different cerebral subdivisions in each species, particularly the mesopallium [16], [29], that were otherwise prone to identification errors with Nissl staining (see anatomy section in methods for a detailed explanation). The results represent hybridizations to over 5,800 serially cut brain sections of entire hemispheres from five species. In species where the brain regions, such as vocal nuclei, are thought to have evolved independently, we used different names following previous designations [7], [30] and the new avian brain nomenclature [29], [31]. Based on their functions, we refer to the ‘posterior vocal pathway’ as a ‘vocal motor pathway’ and the ‘anterior vocal pathway’ as a ‘vocal learning pathway’. However, when we refer to vocal learning nuclei or system, we mean all cerebral vocal/song nuclei in vocal learners, as these nuclei are associated with the presence of vocal learning and also have motor related-neural firing (in songbirds) and/or IEG expression [5]–[7], [32]–[34]. **Figure 2** shows examples of the most reduced movement-associated and singing-driven patterns in songbirds we have obtained. **Table 1** lists all anatomical regions studied and their relative similarities across species.

### Part I. Movement-Associated Brain Areas in Songbirds

#### Migratory restlessness: wing whirring and flights

**Figure 3:**
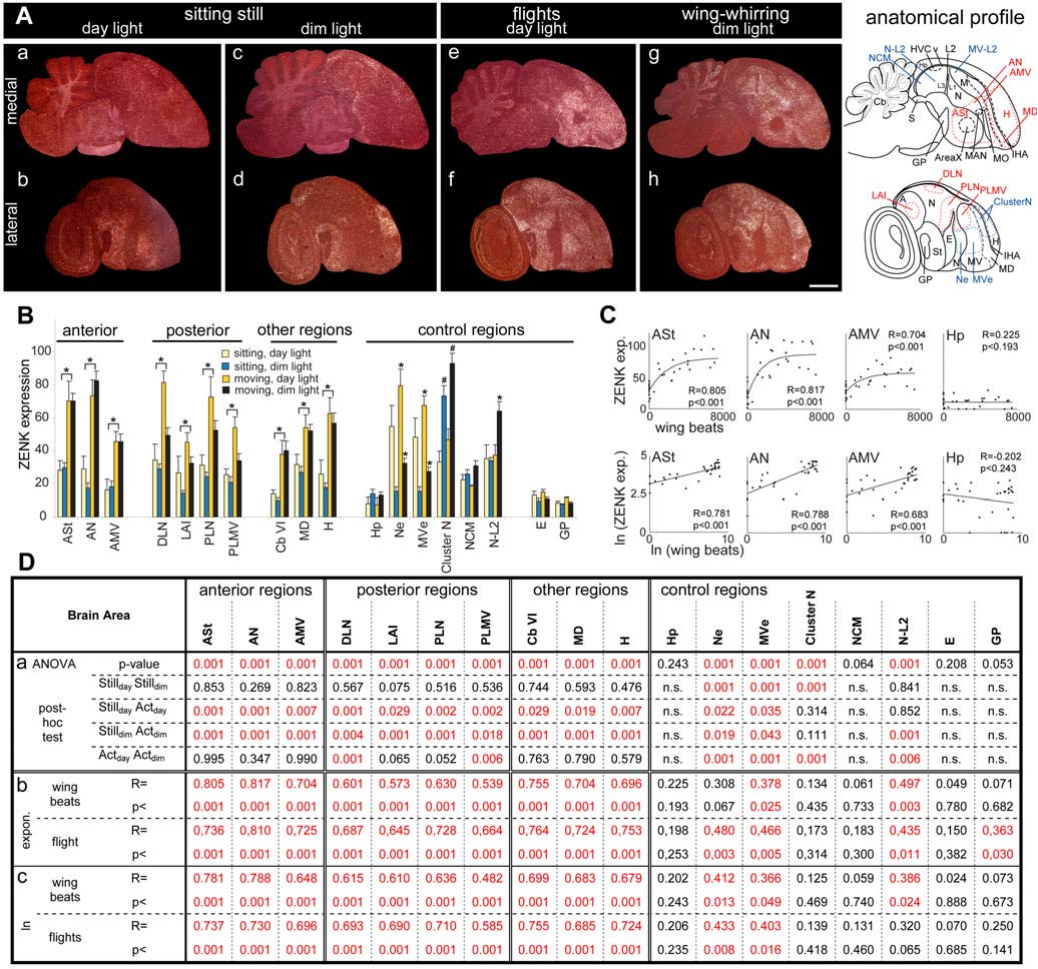
Movement-induced ZENK expression in garden warblers. A. Darkfield images of medial (top) and lateral (bottom row) sagittal sections from birds sitting relatively still in room day light (a, b) or dim night light (c, d) and birds making mostly flights in day light (e, f) or wing whirring in dim light (g, h). The anatomical profiles to the right show the extent of the movement-induced (red) and visual-induced (blue) gene expression areas. Note that the PLN and PLMV areas differ slightly in shape between garden warblers (panel Af,h) and zebra finches (Fig. 2A), due to differences in the shapes of the N and MV cerebral subdivisions and that the warbler sections are more lateral. Anterior is right, dorsal is up. Scale bar, 2 mm. B. Quantification of ZENK expression levels in different brain areas from the four groups of warblers. Anterior and posterior areas were grouped according to their relative location to vocal nuclei. * = p<0.05, one-way ANOVA followed by Holm-Sidak multi-comparison test, comparing moving groups with still groups for each light condition; each movement group showed significant differences with each still group. # = p<0.001 for an increase in Cluster N in dim versus day light groups, whether still or moving. Error bars, S.E.M. C. Correlation between the amount of wing beats and ZENK expression levels shown in exponential (top row) and double natural logarithmic (bottom row) graphs for example areas. Each dot represents the value of one bird. D. Statistical analyses: (a) one-way ANOVA followed by Holm-Sidak all-pairwise multi-comparison test for the brain areas in (B); (b) exponential regression stats (examples in C, top row); (c) linear regression stats on double-logarithmic transformation of the data (C, bottom row). Red text are significant differences (p<0.05); n.s. = not significant.

Migratory restlessness is a highly stereotyped and repetitive behavior performed by migratory songbirds at night in dim light, during their migratory season [35], [36]. We found that under dim light in our cylindrical monitoring apparatus (**Supporting** **Fig. S1A**), migratory songbirds performed migratory restlessness behavior consisting mainly of rapid wing whirring while perched, but also head scans and some hops, in a preferred direction corresponding to the migratory orientation of their free-flying conspecifics [26], [37]. Using infrared video, we quantified wing whirring in garden warblers (*Sylvia borin*) in dim light and flights in day light for 45–60 min. Relative to birds that sat still (**Fig 3A** **a-d**), those that performed flights (**Fig. 3A** **e,f**) or wing whirring (**Fig. 3A** **g,h**) both had high levels of induced ZENK gene expression in the medial part of the anterior cerebrum centered around the anterior vocal nuclei and in areas lateral to the posterior vocal nuclei. The anterior activated areas included the medial part of the anterior striatum (ASt) centered around the striatal vocal nucleus Area X, the anterior nidopallium (AN) centered around the magnocellular vocal nucleus of the anterior nidopallium (MAN, both lateral and medial parts), and the anterior ventral mesopallium (AMV) centered around the mesopallium oval (MO) vocal nucleus; we note here that the MO vocal nucleus is situated in the ventral mesopallium (MV), as revealed by comparisons of GluR1 with ZENK expression (**Fig. S2A**; also seen below for zebra finches and FoxP1 expression). The caudal limits of the anterior activated areas (ASt, AN, and AMV) in birds that made flights formed a semi-linear functional boundary across the respective brain subdivisions (St, N, and MV; **Fig. 3A** **e**). Other activated areas were the medial part of the dorsal mesopallium (MD) and adjacent medial hyperpallium (H; **Fig. 3A** **e, 3B**). The posterior activated areas included the dorsal lateral nidopallium (DLN) adjacent to the HVC vocal nucleus and the lateral intermediate arcopallium (LAI) adjacent to the robust arcopallium (RA) vocal nucleus (**Fig. 3A** **f,h, 3B**; **Fig. S2B** shows higher power images). Expression was also high in the posterior lateral nidopallium (PLN) and adjacent posterior-lateral ventral mesopallium (PLMV; **Fig. 3A** **f,h**) lateral to the two other posterior vocal nuclei-nucleus interface of the nidopallium (NIf) and avalanche (Av) of the ventral mesopallium. However, it was difficult to determine whether NIf and Av were immediately adjacent to the vocal nuclei in garden warblers, as these vocal nuclei are small and more easily identified by singing-driven gene expression (see experiments below with zebra finches).

Although ZENK expression can be found in other brains areas of individual animals or of a group, activation in the brain areas adjacent to the vocal nuclei (3 anterior, 4 posterior) as well as throughout medial MD and medial H occurred whether the birds performed wing movements in day or dim light (**Fig. 3B** **;** statistics in **3Da,** 3^rd^ & 4^th^ rows). In control areas such as the hippocampus and the higher auditory area NCM, there were no increases in expression that were exclusive to the movement groups (**Fig. 3B, 3D** **a**). We also measured two regions as background controls, the entopallium (E, a primary visual region) and the globus pallidus (GP) known not to express high levels of ZENK [6], [38], and found no significant differences among groups (**Fig. 3B, 3D** **a**).

**Figure 4:**
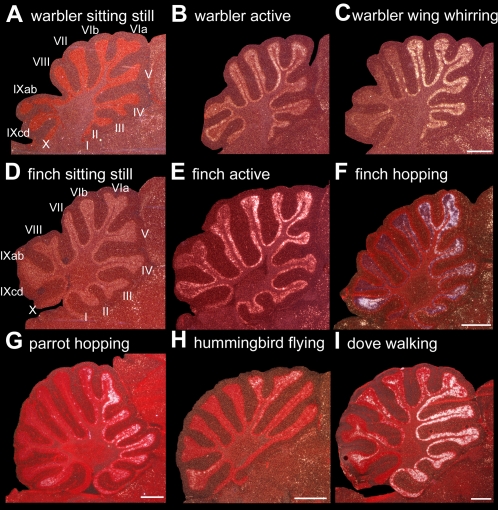
Movement-induced ZENK expression in the cerebellum. Example sagittal sections of: A. Garden warbler sitting still. B. Garden warbler making flights and other movements in day light. C. Garden warbler performing wing whirring in dim light. D. Zebra finch sitting still. E. Zebra finch flying and hopping around the perimeter of the cylinder cage. F. Zebra finch hopping in a rotating wheel. G. Budgerigar hopping in the wheel. H. Anna's hummingbird hovering. I. Ring dove walking on a treadmill. Birds in G and I were deaf; F, G, and I were in the dark. Anterior is right, dorsal is up. Scale bar, 1 mm.

Outside the cerebrum, we noted distinct activation in the granule cell layer of specific cerebellum lobules (**Fig. 4A–C**). Birds that performed flights and were generally active had ZENK activation distributed across lobules I-X (**Figs. 3B** **and** **4B**) albeit with higher levels in lobule VIab (p<0.01, relative to IXcd, paired t-test), whereas birds that performed wing whirring while perched had high activation specific to lobules I-VI (**Fig. 4C**, p<0.012, VIab versus IXcd, paired t-test).

#### Proportionality

In all vocal learning birds, IEG induction levels in vocal nuclei is linearly proportional to the amount of songs produced per 30–60 min and this has been one test to support the conclusion that singing-associated gene expression is motor-driven [5]–[7], [33]. To test for this property in the identified areas, we performed regression analyses on the amount of gene expression with the number of wing beats (counted semi-automatically, both during wing whirring and flights). Similar to the vocal nuclei, the amount of IEG expression in the adjacent anterior and posterior areas, and in the cerebellum, was proportional to the amount of wing beats and flights performed (**Fig. 3C**, top panels **and 3Db**). Interestingly, the relationship was not linear, but was best fitted by a saturating exponential curve (**Fig. 3C** top), similar to the relationship seen in the number of neurons that express ZENK protein in vocal nuclei when birds sing [39]. ZENK mRNA expression levels reached a maximum at ∼6000 wing beats and then saturated, and may have even started to decrease (or perhaps habituate [40]) in some birds. Natural logarithmic transformations of the data were linear (**Fig. 3C**, bottom panels **and 3Dc**), confirming that the original relationship was exponential. In both exponential and logarithmic analyses, the correlations were stronger for the anterior areas and the cerebellum (**Fig. 3D** **b,c**). There were no correlations between wing beats or flights and ZENK expression for the hippocampus, NCM, or the E (**Fig. 3D** **b,c**). A significant but barely detectable correlation with the number of flights was seen in GP, but this is not surprising as the GP is generally known to be involved in motor control [29].

#### Hopping and walking

**Figure 5:**
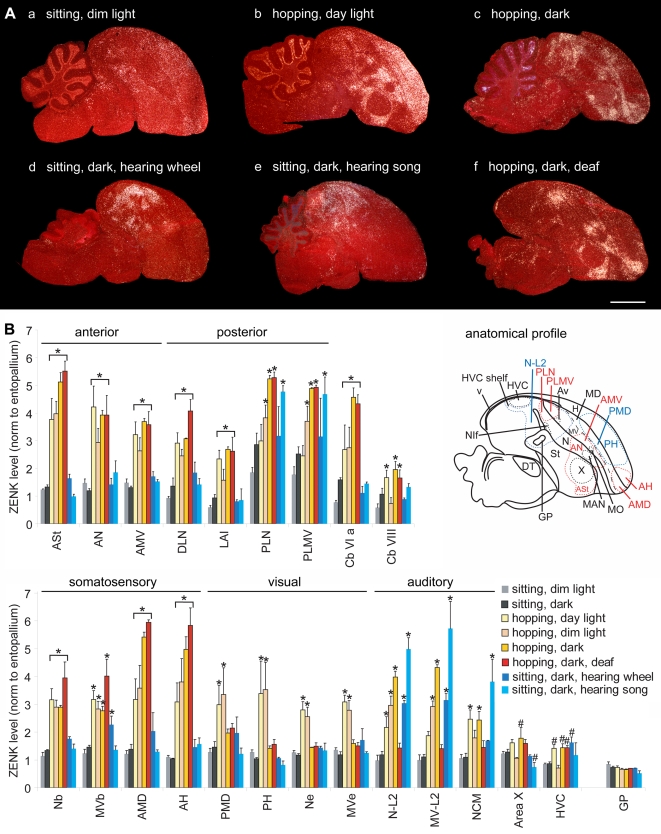
Movement-induced ZENK expression in zebra finches. A. Sagittal sections of birds (a) sitting relatively still in dim light, (b) hopping around the perimeter of a cylindrical cage in day light, (c) hopping in a rotating wheel in the dark, (d) sitting still in the dark and hearing a bird hop in the rotating wheel, (e) sitting still in the dark and hearing playbacks of zebra finch song, and (f) hopping in the rotating wheel in the dark while deaf. The anatomical profile in the lower right highlights the extent of the movement-induced (red) and visual- and auditory-induced (blue) gene expression. Anterior is right, dorsal is up. Scale bar, 2 mm. B. Quantification of ZENK expression levels in 23 brain regions in 8 groups of male zebra finches. Except for a small difference in cerebellum lobule VI, there were no significant differences between sitting still dim light and dark animals; thus, they were treated as one control group for statistical analysis. * = p<0.05 to<0.0001, one-way ANOVA followed by Holm-Sidak multi-comparison test relative to combined values of sitting still animals in dim light+dark (n = 3–6/group). Lines underneath *s indicate brain areas with statistically significant increases exclusive to the moving groups. #s alone indicate values that approached significance by a few tenths of decimal in the ANOVA test. Error bars, S.E.M.

We next analyzed brains of a non-migratory songbird, the zebra finch, which was placed in our cylindrical apparatus. In the day light condition, most zebra finches hopped and walked around the perimeter of the cage at a rapid pace. The ZENK expression pattern in these finches relative to sitting still controls (in dim light, **Fig. 5A** **a**) was similar to that found in the flying day time group of garden warblers, except that the activation of the anterior areas (ASt, AN, and AMV) was more narrowly focused around the anterior vocal nuclei (**Fig. 5A** **b, 5B**). Likewise, the caudal and rostral limits of high expression each lined up to form a strip of activation across the three brain subdivisions: St, N, and MV. Activation in the medial MD and adjacent medial H still spanned the caudal-to-rostral extent of their respective brain subdivisions (**Fig. 5A** **b**). The cerebellum showed a gradient of high expression concentrated towards lobule VI (**Fig. 4E**), similar, but not identical to that seen in garden warblers that performed flights (**Fig. 4B**), and clearly increased relative to finches that sat still (**Fig. 4D** **and** **5B**).

In the above experiments, the movement behaviors were performed voluntarily. Although the birds performed repetitive stereotyped movements, there were still a variety of types across and within animals. To induce a more repetitive single-movement performance, we developed a motorized rotating plexiglass wheel orientated sideways (similar to a hamster wheel, **Fig. S1B**). When the wheel was rotated at a constant rate, in dim light, the zebra finches repeatedly hopped, with a minimum of other movements, to maintain their position at the bottom of the rotating wheel. The ZENK expression pattern in these zebra finches was similar to that in birds that voluntarily hopped around the cage (**Fig. 5B**), though more distinct (shown below in detail for other groups in the wheel) as expected due to mainly one type of movement performed. The activation in the cerebellum was more restricted to the anterior lobules (highest in VI) and in the posterior lobule IXcd (**Fig. 4F**), similar, although not identical to that seen in garden warblers that performed wing whirring behavior (**Fig. 4C**).

#### Visual versus movement activation

In both garden warblers and zebra finches we noted differential activation in other brain areas that varied across groups (**Fig. 3B**, control regions). Some of these areas are parts of known visual pathways. They included: 1) the nidopallium and ventral mesopallium adjacent to the entopallium that we here call Ne and MVe (**Fig. 3A** **f**), which are part of the tectofugal visual pathway and that we previously demonstrated were activated by day-light vision [26]; 2) the posterior part of the medial hyperpallium (PH, also known as the visual Wulst) and the adjacent posterior dorsal mesopallium (PMD; **Fig. 5A** **b**), which are part of the thalamofugal visual pathway [41]; and 3) cluster N, consisting of regions within the most posterior end of the hyperpallium and adjacent dorsal mesopallium (**Fig. 3A** **h**) that we previously demonstrated was activated by dim-light, night-vision in migratory songbirds and implicated in light-mediated magnetic compass detection [26], [42]. We found that cluster N in garden warblers showed strong induced expression by dim light whether the birds sat still or performed migratory restlessness, with no detectable effect of movement (**Fig. 3A** **d,h, 3B, 3Da**). Ne and MVe showed strong induced expression by day light relative to dim light (**Fig. 3A** **b,f, 3B, 3Da**); however, Ne and MVe showed further increased expression in the moving relative to the respective day and dim light control sitting still birds (**Fig. 3B, 3D** **a**), with stronger correlations with the number of flights than with the number of wing beats (**Fig. 3D** **b,c**). Because flights cause optic flow more so than stationary wing whirring movement, we surmise that Ne, proposed to detect visual motion [**43**], as well as MVe, might detect optic flow as the birds move.

**Figure 6:**
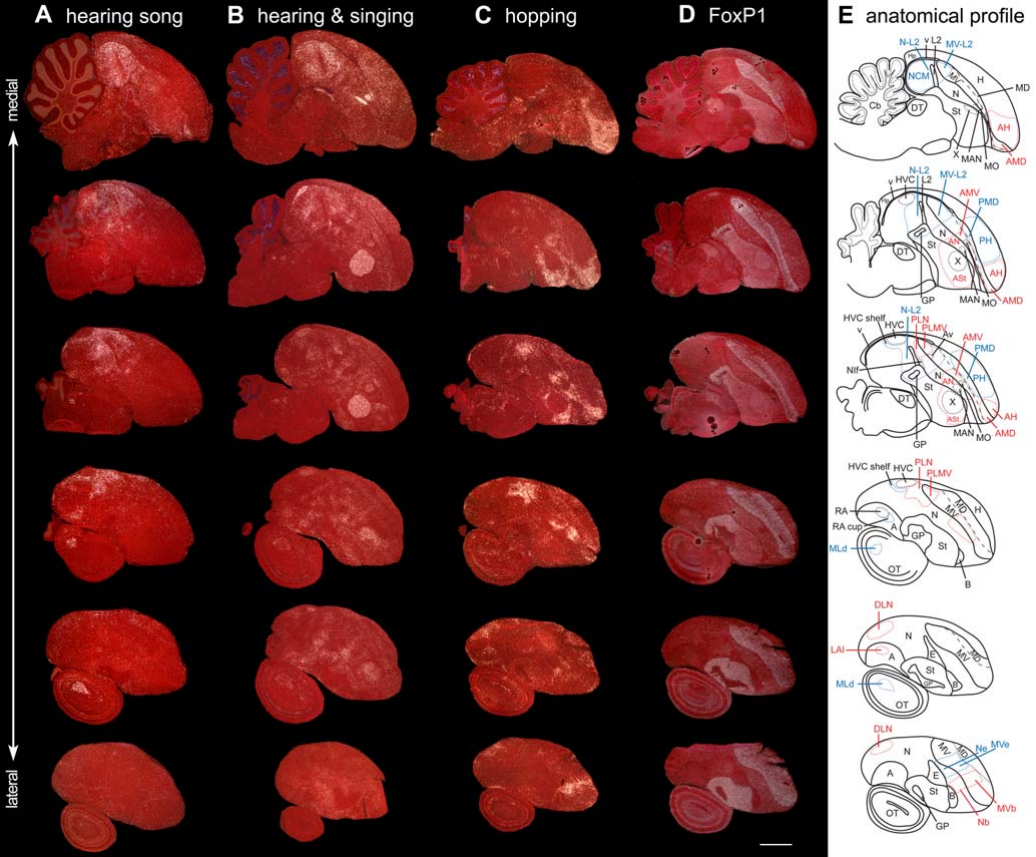
Serial sagittal brain sections of ZENK expression in male zebra finches. A. Auditory areas: bird sitting still in the dark while hearing song. Although not mentioned in the main text, hearing-induced expression also occurs in caudal St and MLd, known auditory regions of the striatum and midbrain, respectively [45]. B. Auditory and vocal areas: bird hearing and singing alone in a sound box in the day light condition. C. Movement areas: bird hopping in the rotating wheel in the dark while deaf. D. FoxP1 expression from adjacent sections of the bird in (C). E. Corresponding anatomical drawings; red: areas with movement-induced expression; blue: areas with auditory- or visual-induced expression. First row are medial-most sections. Anterior is right, dorsal is up. Scale bar, 2 mm. Compare with frontal sections in figure S3.

If visual areas are activated as a result of motion detection, then movement in complete darkness should eliminate this activation. To test this idea, we placed zebra finches in the rotating wheel in complete darkness and observed their movements using an infrared camera. They hopped similarly as the birds moving in dim light. We found that hopping in darkness eliminated the widespread induced expression in visual areas (PMD, PH, Ne and MVe; **Fig. 5A** **c,** **5B;** in-situ shown only for PMD and PH). In contrast, high levels of induced ZENK expression remained in the areas (ASt, AN, and AMV) surrounding the anterior vocal nuclei, the areas (DLN, LAI, PLN, and PLMV) laterally adjacent to the posterior vocal nuclei, known somatosensory areas of the anterior dorsal mesopallium (AMD) and adjacent anterior hyperpallium (AH), and a small strip of cells in the nidopallium (Nb) and ventral mesopallium (MVb) adjacent to the second somatosensory area basorostralis (B; Nb and MVb shown in **Fig. 6C** below, [44]); B like the E does not express high levels of ZENK [38]. High levels of induced ZENK expression also remained in the cerebellum of birds hopping in the dark (**Fig. 5B**). Thus, we conclude that the movement-associated gene expression in the cerebellum, regions of the two known somatosensory pathways, and the regions adjacent to the anterior and posterior vocal pathway nuclei is independent of visual input.

#### Auditory versus movement activation

We noted that in some groups of moving birds there was increased ZENK expression in some of the known auditory areas surrounding the primary auditory field L2 (L2, like the E and B, does not express high levels of ZENK [45]). Such areas include fields L1, L3, and surround (called N-L2 here) and CM (called MV-L2 here), which particularly showed increased ZENK expression in garden warblers that performed wing whirring (**Fig. 3A** **g, 3B, 3Da**) and in zebra finches that performed hopping in the rotating wheel (**Fig. 5A** **c, 5B**), even though the wheel was in a sound isolation box with the motor mounted outside of it (**Fig. S1B**). N-L2 expression in garden warblers had a stronger correlation with the number of wing beats than with the number of flights (**Fig 3D** **b,c**). We surmise that the auditory pathway may be responding to self-produced wing whirring sounds and to hopping sounds on the wheel floor or mechanical sounds transmitted through the rotating wheel, similar to the IEG induction that occurs in N-L2 and other auditory areas when songbirds hear themselves sing [33]. But some reports noted that song playbacks alone can also result in increased ZENK expression in the anterior striatum surrounding Area X, where it has been suggested that the forebrain vocal pathway may be located within an auditory system [46], [47]. This idea was further supported by the facts that HVC sits dorsally adjacent to the HVC shelf and RA caudally adjacent to the RA cup, two known auditory pathway areas with hearing-induced IEG expression [23], [33], [45].

To assess the plausibility of these ideas, we individually placed zebra finches in complete darkness outside of the wheel next to a yoked control bird hopping inside the wheel, of which the former bird could hear the wheel rotate and the bird hop inside of it, but did not move himself and could not see the bird hop. These sitting still, but hearing stimulated birds showed high induced ZENK expression in the N-L2, MV-L2 and other auditory areas, but had no detectable increases in the 11 cerebral regions that include the areas surrounding the anterior vocal nuclei (ASt, AN, and AMV), lateral to the posterior vocal nuclei (DLN, LAI, PLN, and PLMV), and the somatosensory areas (Nb, AMD, AH; except for a small increase in MVb), or in the cerebellum (**Fig. 5A** **d, 5B**).

We also presented birds with playbacks of song while inside our apparatus or the sound isolation chamber. Even in complete darkness, at least half of the birds responded to the song playbacks by hopping excitedly within the confined area, as detected with an infrared camera, and these birds had a similar expression pattern as other hopping birds, including in the areas surrounding the anterior vocal nuclei and lateral to the posterior vocal nuclei (data not shown). In the three birds that remained still, we found robust increased expression in N-L2, including NCM, MV-L2, the HVC shelf, and the RA cup, and other auditory areas as expected [45], but no increased expression in the areas surrounding the anterior vocal nuclei (ASt, AN, and AMV), the areas lateral to the posterior vocal nuclei HVC and RA (DLN and LAI), the somatosensory areas (Nb, MVb, AMD, and AH), or in the cerebellum (**Figs. 5A** **e, 5B, and 6A**). The exception was part of PLN and PLMV; these areas also showed increased robust expression to playbacks of song (**Figs. 5A** **e, 5B, and 6A**).

To further test whether the induced gene expression in these 11 cerebral areas can be independent of auditory input, as is singing-induced gene expression in vocal nuclei [33], we deafened zebra finches and placed them in complete darkness in the rotating wheel. These birds hopped similarly as hearing intact animals. Relative to the hearing intact animals, deafening eliminated the induced expression in all the auditory areas around L2 (N-L2, MV-L2, and NCM), the HVC shelf, and the RA cup (**Figs. 5A** **f, 5B, and 6C**) supporting previous findings that these regions are predominantly auditory [23], [33], [45]. But, these deafened hopping birds still showed strong induced gene expression in the areas surrounding the anterior vocal nuclei (ASt, AN, and AMV), in all four areas lateral to the posterior vocal nuclei (DLN, LAI, PLN, and PLMV), the somatosensory areas (Nb, MVb, AMD, and AH), and the cerebellum (**Figs. 5A** **f, 5B, and 6C**); this is the most distinct movement-associated expression pattern that we have seen. We conclude that the induced expression in the auditory areas of moving animals is a result of the animals hearing movement- and/or mechanically-generated sounds, and the remaining areas have movement-associated gene expression that is independent of auditory input. We also conclude that the same parts of PLN and PLMV can independently have movement-induced and hearing-induced gene expression.

We noticed that although ZENK expression in vocal nuclei was much lower than in the adjacent brain areas of the moving birds, in some animals there were some cells that expressed ZENK (**Fig. 5A** **b,c,f**). Thus, we quantified ZENK expression in Area X and HVC of all birds and found a trend of low-level increased expression that approached significance in some of the moving groups (**Fig. 5B**). Future experiments will be needed to determine if this increase is real and associated with limb and body movements or whether some birds produce some vocalizations while hopping.

#### Physical relationship to vocal nuclei

**Figure 7:**
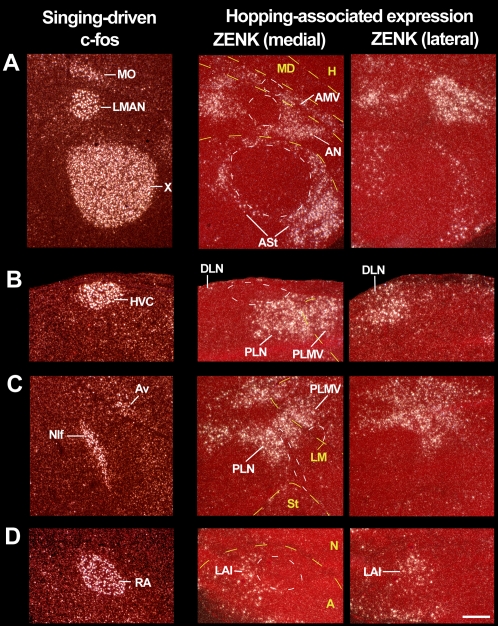
High power images of IEG activation in zebra finch vocal nuclei during singing and in adjacent movement-associated areas during hopping. (A) Anterior vocal nuclei adjacent to ASt, AN, and AMV. (B) HVC adjacent to DLN and dorsal PLN. (C) NIf and Av adjacent to ventral PLN and PLMV respectively. (D) RA adjacent to LAI. The c-fos expression in vocal nuclei (first column) is of a young zebra finch male that sang for 30 min while moving relatively little; c-fos is shown for its high contrast in vocal nuclei relative to the surrounding non-vocal areas. The hopping-associated expression (left two columns) is from a male that hoped in the dark and was deaf; the left most sections are lateral to the vocal nuclei (except for anterior areas, which still contain the lateral part of the anterior vocal nuclei). Yellow dashed lines-brain subdivision boundaries; white dashed lines–vocal nuclei boundaries, only highlighted for some images so that other sections can be viewed as is. Anterior is right, dorsal is up–sagittal sections; sections of the top panel are orientated at a ∼45° angle so that all three anterior vocal nuclei fit vertically into one image. Scale bar, 200 µm.

To determine how close the movement-associated areas are to the vocal nuclei and to clarify the relative physical relationship of the posterior vocal nuclei to auditory areas, we quantified the distances in Nissl stained serial sagittal (**Figs. 6** **and** **7**) and coronal (**Fig. S3**) sections of the deaf, dark, hopping animals. We used serial sections of hearing song-induced and singing-induced IEG expression in other birds, along with FoxP1 expression in adjacent sections, to help identify the auditory regions, the vocal nuclei, and cerebral subdivision boundaries (**Figs. 6** **and** **7** **;** **Fig. S3** **;** see methods). The ZENK activated neurons of ASt, AN, and AMV were on average only ∼11, 28, and 10 µm distant from the edge of vocal nuclei Area X, LMAN, and MO respectively (range 0–30 µm, **Table 2**). That is, there were either no or 1–3 unlabelled neurons between the movement-activated neurons and these vocal nuclei. This expression within ASt, AN, and AMV surrounded the central portions of the anterior vocal nuclei, was most prominent anterior and ventral to them (**Fig. 7A** **,** medial), and was less on their caudoventral sides further laterally (**Fig. 7A** **,** lateral). This pattern suggests a topographical relationship in the functional activation across brain subdivisions.

**Table 2:** Distances between movement-associated areas and vocal nuclei

Areas	Distance µm±SEM
ASt-AreaX	11.66±0.96
AN-MAN	28.33±5.09
AMV-MO	10.55±1.54
DLN-HVC	88.89±11.06
PLN-HVC	10.00±0
PLN-NIf	8.61±2.47
PLMV-Av	17.22±5.47
LAI-RA	28.89±10.02
Nb-MAN	962.50±23.94
MVb-MO	1,167.00±33.33

For the posterior areas, the dorsal part of PLN was ∼10 µm ventral and anterior to the lateral ∼1/3^rd^ of HVC (**Fig. 7B** **;** brightfield in **Fig. S4Ab**), whereas DLN began ∼88 µm posterior to HVC and increased in size further laterally and posteriorly (**Fig. 7B** **;** **Fig. S4Ac** **;** coronal in **Fig. S3Ba**). The auditory shelf directly ventral to the medial ∼2/3^rds^ of HVC that shows auditory-induced expression (**Fig. 6A** **,** 3^rd^ and 4^th^ row) did not show any clusters of labeled neurons in the hopping deaf animals (**Fig. 6C** **;** **Fig. S4Aa**). For the location of PLN and PLMV relative to NIf and Av, it was simple to identify the vocal nuclei in singing animals using IEG expression (**Fig. 7C**), but difficult to identify by Nissl alone in the hopping animals. However in the hopping animals, we noted a split of expression within PLN where NIf (but also adjacent field L2) would be located and a split between PLN and PLMV by the mesopallium lamina (LM, **Fig. 7C** **,** medial**;** **Fig S4Bb**). To determine if the first split was due to NIf, we false colored the IEG expression from singing and hopping deaf animals, aligned them by the LM boundary, and found that PLN appeared to surround the dorsal ∼1/3^rd^ of NIf and that PLMV was directly dorsal to Av (**Fig. S4D**). Further lateral, when NIf was no longer present, the split in PLN was no longer present (**Fig. 7C** **,** lateral**;** **Fig. S4Bc**). Further medial, field L2 was easily seen by its smaller size neurons relative to L1 and L3, but there was no movement-associated expression around it (**Fig. S4Ba**). Using these alignments, we calculated that PLN and PLMV are on average ∼8 and 17 µm from the NIf and Av respectively (**Table 2**). For LAI, it was ∼28 µm caudal to the lateral ∼1/3^rd^ of RA (**Fig. 7D** **,** medial**;** **Fig. S4Cb** **,** **Table 2**), and wrapped around RA laterally such that it occupied a similar central position in the arcopallium (**Fig. 7D** **,** lateral; **Fig. S4Cc** **;** coronal in **Fig. S3Bb**). There was also some expression in the arcopallium caudoventral to RA (**Fig. 7D** **;** **Fig. S3Bb**). The auditory RA cup anteroventral to the medial ∼2/3^rds^ of RA that shows hearing-induced expression (**Fig. 6A** **,** 4^th^ row) did not show detectable clusters of movement-activated neurons (**Fig. 6C** **;** **Fig. S4Ca**). In contrast to these distances, the known somatosensory areas with movement-associated expression (Nb and MVb) were at a minimum ∼962 and ∼1,167 µm distant to their nearest vocal nuclei MAN and MO, respectively (**Table 2**). We did not measure distances for the other two known somatosensory areas (AMD and AH), as there are no known vocal nuclei in their respective brain subdivisions (MD and H). Based on these results, it is clear that 7 of the 11 movement-associated cerebral areas are directly adjacent to the 7 known vocal nuclei; the remaining 4 brain areas not adjacent to the vocal nuclei are known somatosensory regions.

#### Relative cerebral volume

**Figure 8:**
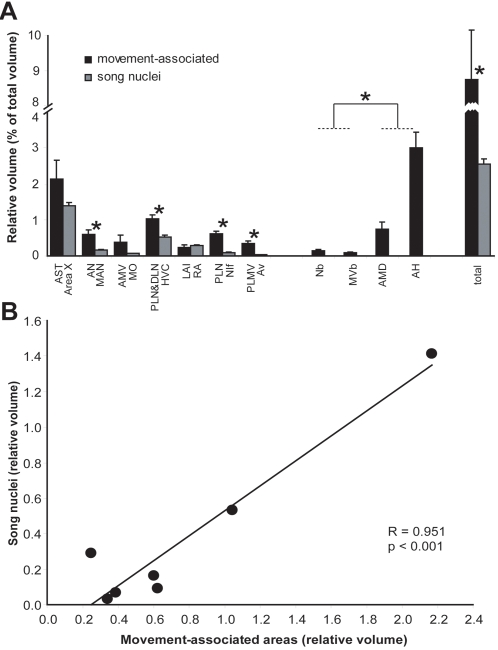
Relative volumes of movement-associated areas and vocal nuclei. A. Relative volumes as a percentage of the summed cerebral (telencephalon) volume from a series of sagittal sections (see methods) from the hopping, dark and deaf animals. The movement-associated areas and adjacent vocal nuclei are shown as bars adjacent to each other. Totals represent the summed relative volumes of all movement-associated areas whether or not it is adjacent to a vocal nucleus, and of all vocal nuclei. * = p<0.05, paired t-test (within bird comparisons, n = 3). Error bars, S.E.M. Although all animals had higher volumes of ASt and AMV relative to Area X and MO, the variance was large in the movement areas such that the volume difference did not reach significance. B. Correlation between relative volumes of movement-associated regions and adjacent vocal nuclei. Each dot represents the average values from the graph in (A).

We next made a gross assessment of the relative cerebral volume activated during hopping. We found that the movement-associated areas in the deaf, dark, hopping animals comprised ∼8.7% of the cerebrum volume (**Fig. 8A**, total). The largest contributor to this volume was the ASt around Area X and the somatosensory AH within the hyperpallium (**Fig. 8A**). In comparison, the song nuclei combined comprised ∼2.5% of the cerebrum volume, with Area X the largest contributor (**Fig. 8A**). Most of the movement-associated areas were proportionally larger than the adjacent vocal nuclei; LAI and RA were the exceptions, which were about the same size. In this regard, there was a significant positive correlation between vocal nuclei size and adjacent movement-associated region size (**Fig. 8B**). This distinct hopping-induced expression pattern contrasts with the pattern found in birds taken from our aviary in the early morning, after 60 min of lights, hearing songs and calls, feeding, flying, hopping, and making physical contact with other birds, which results in widespread ZENK expression in the cerebrum (**Fig. S5A**). Based on these results, it becomes clear that the hopping-induced expression pattern comprises distinct domains of the cerebrum (**Fig. 6C**), most of which are directly adjacent to the vocal nuclei.

#### Confirmation with another IEG

**Figure 9:**
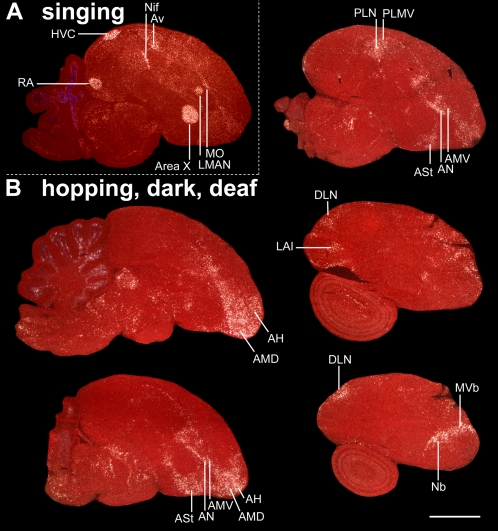
Movement-induced c-fos expression in zebra finches. A. Vocal areas: brain section containing all seven cerebral vocal nuclei from a singing bird. B. Movement areas: serial sections from a hopping bird in the rotating wheel in the dark while deaf. The patterns are similar to that found with ZENK (Fig. 6C). See figure 6E for delineation of anatomical boundaries. Anterior is right, dorsal is up. Scale bar, 2 mm.

We tested whether movement-associated gene expression was specific to ZENK or a general IEG property, by examining expression of another IEG, c-fos, which is generally thought to be less sensitive to small changes in neural activity, and thus can reveal areas that were highly active during a specific behavior. We found that c-fos was induced in the same cerebral brain areas adjacent to the vocal nuclei as well as in known somatosensory areas (**Fig. 9**), but with a higher contrast of activation relative to ZENK (**Fig. 6C**). As with ZENK, the c-fos induction in these dark and deaf animals was independent of visual and auditory input.

Summarizing Part I of this study, in songbirds, 11 cerebral areas showed movement-associated activation that can be grouped into 4 clusters: an AH and AMD cluster comprising known subdivisions of a somatosensory pathway; a Nb and MVb cluster within known subdivisions of a second somatosensory pathway; an anterior ASt, AN, and AMV cluster surrounding the three anterior vocal pathway nuclei Area X, MAN, and MO, respectively; and a posterior DLN, LAI, PLN, and PLMV cluster posterior-laterally adjacent to the four posterior vocal pathway nuclei HVC, RA, NIf, and Av respectively. We do not know if the movement-associated IEG activation adjacent to the vocal nuclei is the result of pre-motor activity as occurs in the vocal nuclei during singing [32]–[34], is the result of somatosensory feedback activity via muscle spindles, or is from a combination of both; thus, we call the induced gene expression as movement-associated. Regardless of the specific source of activation, these findings led us to hypothesize that vocal learning systems may have evolved out of a pre-existing motor or somatosensory-motor system, which may explain the anatomical similarities among distantly related vocal learning birds. If true, then the other vocal learning birds should show a similar relationship between movement-associated areas and their cerebral vocal nuclei, an idea we tested next.

### Part II. Movement-Associated Brain Areas in Other Vocal Learners

#### Parrots

**Figure 10:**
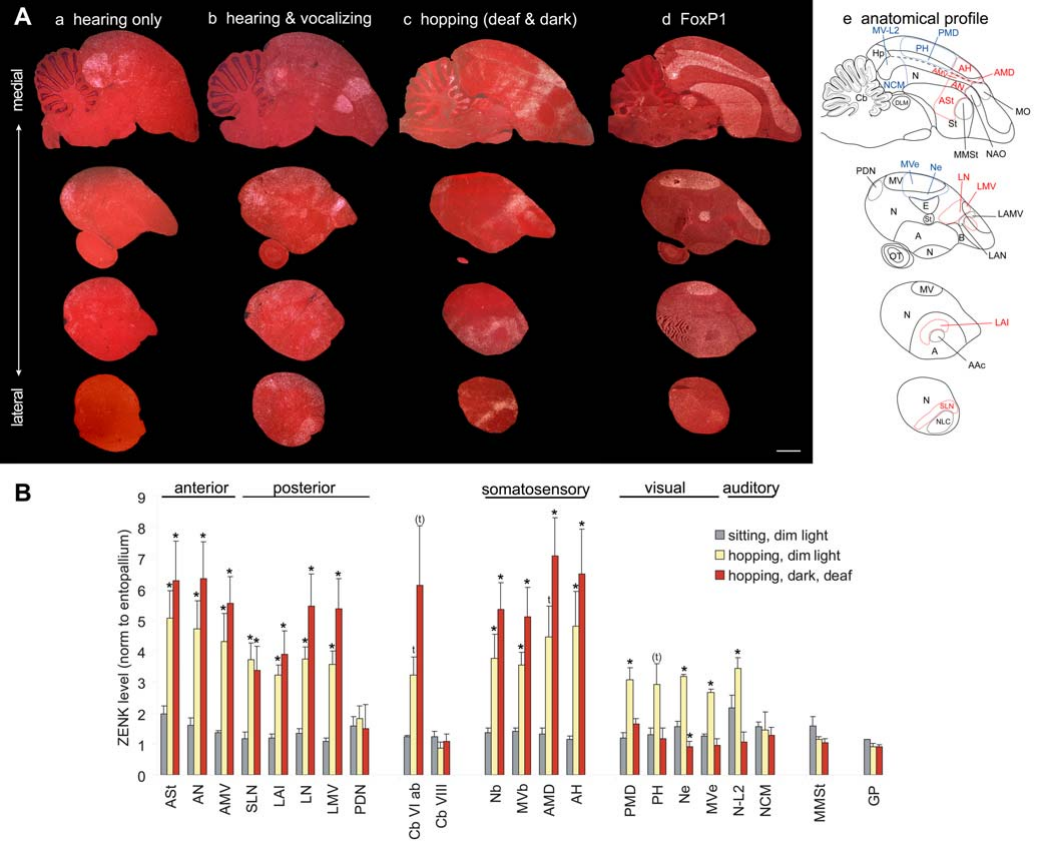
Movement-induced ZENK expression in budgerigars, a parrot. A. Serial sagittal sections of: (a) Auditory areas: bird sitting relatively still while hearing playbacks of budgerigar warble song; (b) Auditory and vocal areas: perched bird hearing himself and producing warble song while alone and moving relatively little; (c) Movement areas: bird hopping in the rotating wheel in the dark while deaf; (d) FoxP1 expression from adjacent sections of the bird in (c); (e) Corresponding anatomical drawings; red: areas with movement-induced expression; blue: areas with auditory- or visual-induced expression. First row are medial-most sections. Sections with N-L2 are not shown. Anterior is right, dorsal is up. Scale bar, 2 mm. Compare with frontal sections in figure S6. B. Quantification of ZENK expression levels in 22 different brain regions in three groups of budgerigars. * = p<0.05 to<0.0001, one-way ANOVA followed by Holm-Sidak multi-comparison test relative to the sitting still group (n = 3/group). t = significantly different, p<0.05, by a t-test. (t) = 0.06<p<0.09 by a t-test. Although the cerebellum lobule VI clearly had high levels of induced expression in all three animals of the hopping, dark, deaf group, this difference approached significance (p = 0.06) due to a large variance; the same was true of the PH region in the hopping, dim light group. Error bars, S.E.M.

In parrots, the three anterior vocal pathway nuclei are situated in nearly identical brain locations as their analogous counterparts in songbirds, but the counterparts of the four songbird posterior vocal nuclei are situated much further (1000s of µm) laterally away from the main avian auditory pathway areas (**Fig. 1B** **,** yellow). Therefore, this differential relationship provides a natural experiment to test whether movement-associated brain areas would be adjacent to parrot vocal nuclei or instead in the same location as in songbirds, near the auditory pathway. To perform this test, we placed deafened budgerigars (*Melopsittacus undulatus*, a small parrot) in the rotating wheel in complete darkness. The parrots performed hopping movements similar to zebra finches, and in addition often bobbed the head before a hop. We compared brain ZENK expression in these birds with sitting or hopping animals in dim light, and with hearing intact animals that either heard or produced parrot warble song (**Fig. 10A** **a,b**) [6].

Similar to deafened songbirds hopping in darkness, the induced ZENK and c-fos expression patterns in the deafened parrots hopping in darkness were distinct, with the highest levels distributed across at least nine cerebral brain areas of which seven were directly adjacent (within 100 µm) to the seven parrot vocal nuclei (**Fig. 10A** **c, 10B;** c-fos not shown). **Figure S6** shows coronal sections for comparison. These brain areas were: ASt, AN, and AMV with the highest expression directly caudal and medial to the parrot anterior vocal nuclei MMSt, NAO, and MO (analogs of songbird Area X, MAN, and MO respectively); the lateral nidopallium (LN) surrounding LAN (proposed analog of songbird NIf) and the adjacent lateral ventral mesopallium (LMV) surrounding LAM (proposed analog of songbird Av); the LAI surrounding AAC (analog of songbird RA); and the supra-lateral nidopallium (SLN, for details see anatomy section in methods) surrounding NLC (analog of songbird HVC) (**Fig. 10A** **c, 10B;** **Fig S6B**). For each of these posterior areas (LN, LMV, LAI, and NLC), expression was highest dorsally and/or posteriorly to the vocal nuclei. Also as in songbirds, the somatosensory areas of AMD and AH showed robust induced expression (**Fig. 10A** **c,** 1^st^ row **and 10B;** **Fig S6B** **,** 1^st^ row). There was activation in the presumed second somatosensory areas Nb and MVb adjacent to B, but these two areas are also adjacent to two of the posterior vocal nuclei of parrots (LAN and LAM) making it difficult to parse out the boundaries, if any (**Fig. 10A** **c,** 2^nd^ row **and 10B;** **Fig S6B** **,** 2^nd^ row). There were no detectable high levels of induced expression in the known auditory areas (N-L2, NCM) or in the posterior dorsal nidopallium (PDN) where songbird DLN and HVC would be expected to be located (**Fig. 10A,B**) if they were in the same relative location to the auditory pathway as in songbirds. The anterior vocal nuclei, such as MMSt also did not show induction (**Fig. 10A** **c, 10B**). In hearing intact hopping budgerigars in dim light, induced expression occurred in the same brain areas as well as in visual (PMD, PH, Ne, and MVe) and auditory (N-L2) areas (**Fig. 10B**) similar to that found in songbirds, presumably due to optic flow in dim light and to the animals hearing the hops and/or the mechanical rotating sounds of the wheel, respectively. In both moving groups, there was induced expression in the anterior half of the cerebellum (highest in VI) and in lobule IXcd (**Figs. 4G** **and** **10B**), similar to that seen in zebra finches that hopped in the wheel (**Fig. 4F**).

#### Hummingbirds

We next investigated the Anna's hummingbird (*Calypte anna*), a known vocal learner [48]. The three anterior vocal pathway nuclei of hummingbirds are situated in nearly identical brain locations as in songbirds and parrots, but the proposed counterparts of the four posterior vocal nuclei are situated at intermediate caudal-lateral locations, partly adjacent to the auditory pathway areas (**Fig. 1B** **,** yellow). Attempting to perform similar movement experiments with hummingbirds would not work, because hummingbirds rarely walk or hop for mobility. Instead, they fly, even to move a few centimeters from one perch location to another. Further, when Anna's hummingbirds were placed in darkness for 2–3h to reduce brain IEG expression to basal levels, they went into torpor, a unique hibernation-like sleeping state that makes them immobile [49]. Thus, for the hummingbirds, we plugged one ear with clay and covered the ear and ipsilateral eye with black vinyl tape to reduce visual motion- and auditory wing humming-induced gene expression in one hemisphere. Covering one eye in songbirds causes ZENK expression to be reduced in visual pathways of the contralateral hemisphere [27] (Hara, Kubikova, Hessler, Jarvis; submitted), as in laterally eyed birds the visual pathways are nearly completely crossed [50]. Auditory input is bilateral, but removing input from one ear in chicks causes some auditory nuclei to have reduced 2-deoxyglucose activation in the contralateral hemisphere [51], [52]. We then placed the hummingbirds in dim light inside a plexiglass box with a central perch (see methods). Most Anna's hummingbirds remained relatively still on the perch for the first several hours; thereafter, some birds performed stereotypical circular hovering flights on and off the perch in the direction of the open eye while others remained perched, relatively still (less than ∼15 flights in 60 min), and awake.

**Figure 11:**
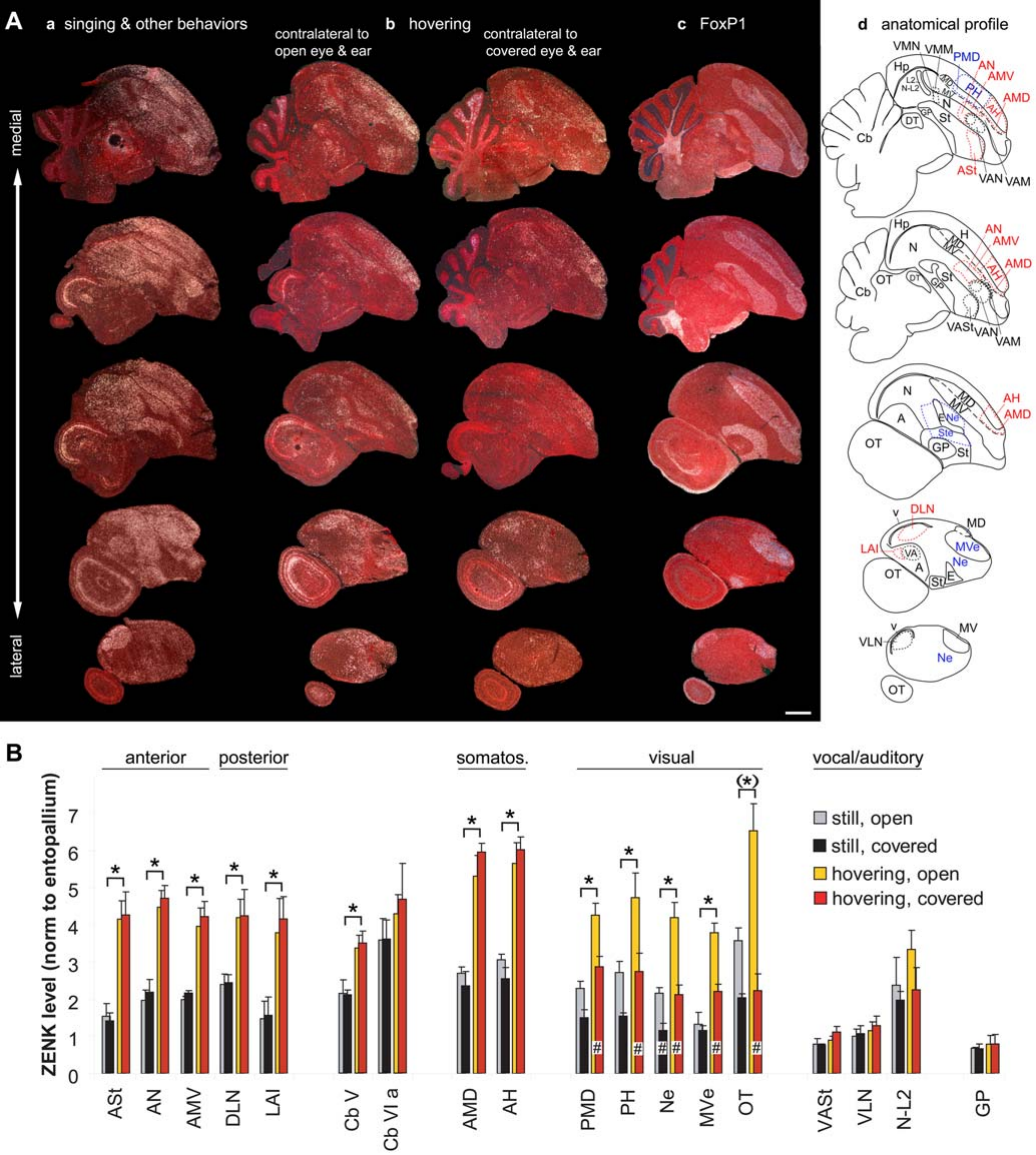
Movement-induced ZENK expression in male Anna's hummingbird. A. Serial sagittal sections of: (a) Vocal and other areas: ZENK expression in a bird that was singing interspersed with flying near an outdoor feeder in the morning (high expression in non-vocal areas is due to other behaviors, including flying and feeding); (b) Auditory, visual, and movement-associated areas: ZENK expression in control hemisphere (contralateral to open eye and ear) and experimental hemisphere (contralateral to covered eye and ear) of a bird hovering in a plexiglass cage in dim light; (c) FoxP1 expression from adjacent sections of the experimental hemisphere of the bird in (b); (d) Corresponding anatomical drawings; red: areas with movement-induced expression; blue: areas with visual- or auditory-induced expression (auditory areas also determined from a previous study [7]). First row are medial-most sections. Anterior is right, dorsal is up. Scale bar, 2 mm. Compare with coronal sections in figure S7. B. Quantification of ZENK expression levels in 18 different brain regions, in both hemispheres, in two groups of hummingbirds. * indicates brain areas with statistically significant increases in both experimental (covered) and control (open) hemispheres of hovering birds relative to the experimental and control hemispheres of the relatively still birds (p<0.05, one-tailed t-test, n = 3 relatively still and 4 hovering animals for both hemispheres). # indicates significantly less increase in the experimental hemisphere (p<0.05, paired t-test on experimental and control hemispheres within birds). The (*) for the OT indicates that this is the only visual area that did not show increased ZENK expression in the experimental hemisphere opposite the covered eye. Error bars, S.E.M.

**Figure 12:**
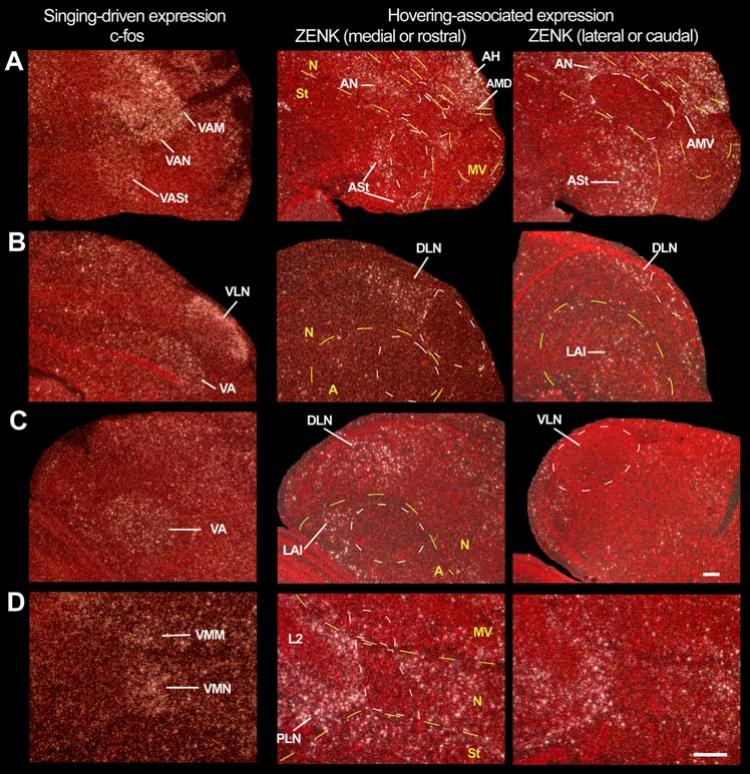
High power images of IEG activation in hummingbird vocal nuclei during singing and in adjacent movement-associated areas during hopping. A. Anterior vocal nuclei adjacent to ASt, AN, and AMV in sagittal sections. B. DLN adjacent to VLN [and LAI to VA] in coronal sections. C. LAI adjacent to VA [and DLN to VLN] in sagittal sections. D. VMN [as well as VMM] adjacent to activated areas near L2 in sagittal sections. The c-fos expression in vocal nuclei (first column) is of male that sang for 30 min interspersed with flying and feeding; c-fos is shown for its high contrast in vocal nuclei relative to the surrounding non-vocal areas. The hovering-associated expression patterns (left two columns) are from the hemisphere opposite of the covered eye and ear of males that hovered in a plexiglass cage. Anterior is right, dorsal is up for sagittal sections; medial is left, dorsal is up for frontal sections. The left most sections are either lateral (A, C, and D) or caudal (B) to that shown in the middle column. Different background red color is due to different cresyl violet staining intensities. Note that similar to budgerigar MO and NAO (Fig. 10Ab and Fig. S6A), the two analogous hummingbird pallial anterior vocal nuclei (VAM and VAM) are very close such that the IEG expression does not distinguish the brain subdivision boundary well. Yellow dashed lines-brain subdivision boundaries; white dashed lines–vocal nuclei boundaries, only highlighted for some images so that other sections can be viewed as is; boundaries were determined from Nissl stain and adjacent sections hybridized with FoxP1 (Fig. 11Ac and Fig. S7C). Scale bars, 200 µm.

In the hovering hummingbirds, there was an induced ZENK expression pattern that was discrete and significantly different between hemispheres, which allowed us to suggest which brain areas have movement-associated versus visual- and possible auditory-associated gene expression (**Fig. 11A** **b**). **Figure S7** shows serial coronal sections for comparison. Apparent visual pathway areas (PMD, PH, Ne, MVe, and optic tectum [OT]) showed increased expression in the animals that made hovering flights, but this expression was significantly reduced in the hemisphere contralateral to the covered eye (**Fig. 11A** **b, 11B;** **Fig. S7B**). A similar and visible, but non-significant trend was seen in the auditory region N-L2 (including NCM; **Fig. 11A** **b, 11B;** **Fig. S7B**), perhaps due to crossed pathways. In contrast, there was equivalent bilateral increased expression in hovering birds within the ASt, AN, and AMV medial to and surrounding the anterior vocal pathway nuclei VASt, VAN, and VAM (analogs of songbird Area X, MAN, and MO, respectively), in the DLN medially adjacent to the VLN vocal nucleus (analog of songbird HVC), and in the LAI caudally and ventrally adjacent to the VA vocal nucleus (analog of songbird RA; **Figs. 11A** **b, 11B**; **Fig. S7B** **;** higher power images shown in **Fig. 12A–C**). Although hummingbird DLN expression was distinctly medially adjacent to VLN instead of lateral to it as in songbirds, the hummingbird VLN is positioned more laterally than the songbird analog HVC and thus the DLN area of movement-associated activation is in a similar location as that of songbirds (**Fig. 12B** **vs** **Fig. S3Ba**). As in songbirds and parrots, these areas of apparent movement-associated gene expression were less than 100 µm distant to the vocal nuclei.

There was also increased bilateral expression in the apparent somatosensory areas AMD and adjacent AH (**Fig. 11A** **b** top three rows, **11B and 12A**). We did not note high expression levels in the second somatosensory areas Nb and MVb. We could not reliably determine whether there was induced expression adjacent to the other two posterior vocal nuclei VMN and VMM (proposed analogs of songbird NIf and Av) in the medial nidopallium and mesopallium respectively, because these nuclei in hummingbirds, more so than in songbirds, are relatively small and difficult to locate without singing-induced gene expression; likewise, they are also directly adjacent to auditory areas. Nevertheless, in the several birds where we were able to locate these nuclei, there was still adjacent high induced ZENK expression bilaterally, but in regions that, like songbird PLN and PLMV, also show hearing-induced gene expression (**Fig. 11A** top row, **and 12D** [7]). The cerebellum had increased activation in lobules V and IXcd (**Fig. 4H**), similar, although not identical, to the pattern seen in garden warblers that performed wing whirring (**Fig. 4C**); hummingbirds have under-developed lobules II and III, which are thought to modulate leg movements [53] (but see discussion). Lobule VI already had high expression in the relatively still animals that performed few flights (**Fig. 11B**).

Summarizing part II of this study, all seven parrot and at least most hummingbird cerebral vocal nuclei are directly adjacent to areas that are activated during movement, even though some of the posterior vocal pathway nuclei are in different brain locations in each group. This activation does not require visual or auditory input. These findings suggest that vocal learning systems in distantly related birds are adjacent to a pre-existing system involved in movement control. If true, then vocal non-learning birds should have similar brain areas associated with movement control, an idea that we tested next.

### Part III. Movement-Associated Brain Areas in Vocal Non-Learners

#### Female songbirds

Females of many songbird species, including zebra finches, do not have vocal learning behavior, i.e. song or learned calls [12]. Their forebrain vocal nuclei (except for LMAN) are atrophied as seen in zebra finch females [54] and garden warblers (noted here). We separated out the females in the garden warbler groups described above (n = 10) and found that in those that performed movement behaviors (wing whirring and flights) ZENK gene activation was present within comparable cerebral areas and in the cerebellum as seen in males, but without the presence of negative expression regions of vocal nuclei (**Fig. S8A** **,** except for LMAN). To determine a more restricted pattern, we analyzed ZENK expression in the brains of deafened female zebra finches placed in the rotating wheel in the dark. Movement-induced ZENK expression was found in the same 11 cerebral brain areas and in the cerebellum as in males, except that the expression where the anterior vocal nuclei would be expected to be located was patchier and diffuse (**Fig. S8B** **;**PLN, PLMV, and LAI not shown; p<0.02 hopping females [n = 4] relative to sitting males [n = 6]; p>0.05 hopping females [n = 4] relative to hopping males [n = 3] for all 11 areas, two-tailed t-test). Thus, these movement-associated areas appear to be present independent of the presence of functioning vocal nuclei. However, female songbirds of an ancestral species may have once had vocal learning behavior and associated brain nuclei that were then subsequently lost in some species. Thus, to test our hypothesis further, we examined movement-associated gene expression in a vocal non-learning species.

#### Ring Doves

**Figure 13:**
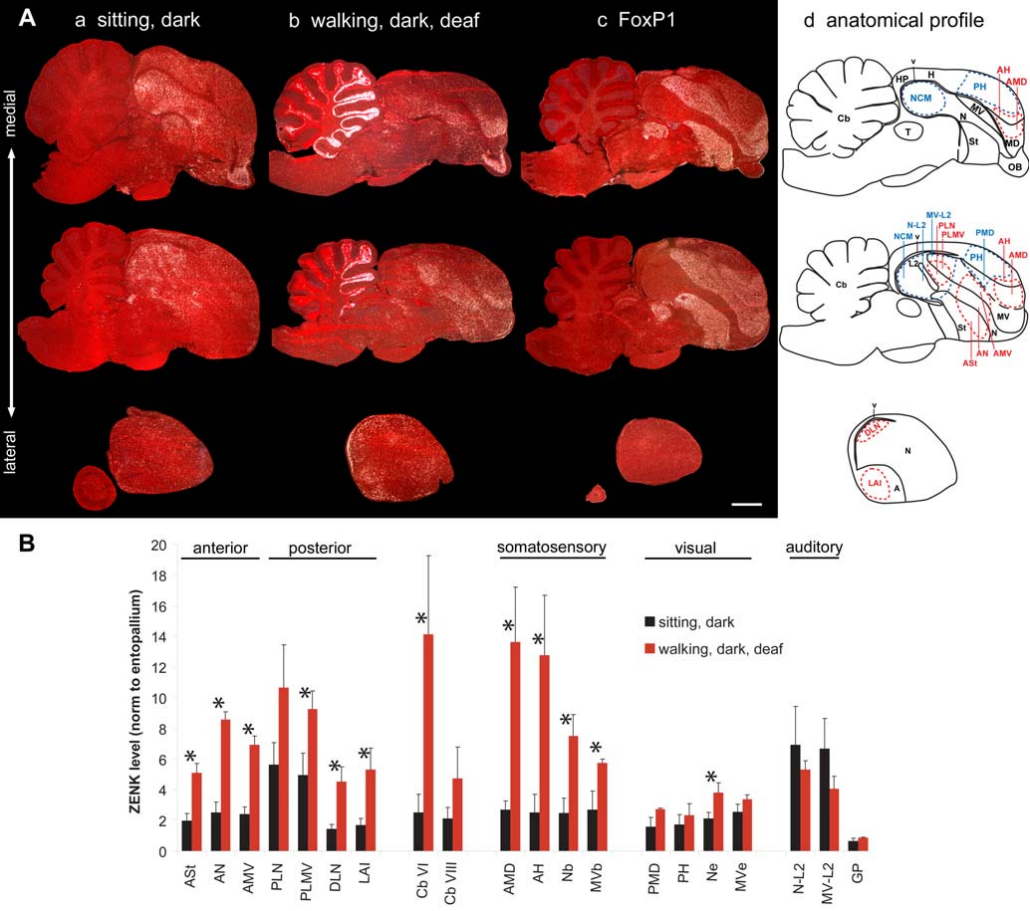
Movement-induced ZENK expression in ring doves, a vocal non-learner. A. Sagittal sections of: (a) Dove sitting relatively still in the dark; (b) Dove walking on a treadmill in the dark while deaf; (c) FoxP1 expression from adjacent sections of the bird in (b); (d) Corresponding anatomical drawings; red: areas with movement-induced expression; blue: known auditory and visual areas. First row are medial-most sections. Front is right, dorsal is up. Scale bar, 2 mm. B. Quantification of ZENK expression levels in 20 different brain regions in two groups of ring doves. * = p<0.05 to<0.0001, one-tailed t-test, relative to sitting still animals (n = 3/group). Error bars, S.E.M.

Ring doves (*Streptopelia risoria*) are interrelated between songbirds and hummingbirds (**Fig. 1A**). Nevertheless, they are known vocal non-learners [55] and do not possess cerebral vocal nuclei [15]. As ring doves are bigger than zebra finches and budgerigars, they did not fit into our rotating wheel apparatus. Further, ring doves do not normally hop when they move from one nearby location to another, but instead walk. Therefore, we placed deafened ring doves in darkness on a treadmill designed for animals the size of rats and compared their gene expression to intact controls that sat still (**Fig. 13A,B**).

We found that in sitting controls, there was low ZENK expression throughout the brain except in the auditory pathway, which had unusually high basal levels that were partly reduced by deafening (**Fig. 13A** **a,b**). The olfactory bulb also had high basal levels. When the treadmill floor moved, the doves walked. ZENK expression in these doves was induced to high levels in anterior cerebellum lobules I-VI and posterior lobule IXcd (**Fig. 4I**), similar, although not identical to that seen in songbirds and budgerigars that hopped (**Fig. 4F, G**). In addition, there was increased expression in somatosensory areas of AMD and adjacent AH, and Nb and adjacent MVb (**Fig. 13A** **b, 13B**; but more widespread in Nb and MVb, not shown) similar to that seen in songbirds, parrots, or hummingbirds (except for Nb and MVb in the latter). When we examined expression in the anterior medial forebrain where we would expect to find anterior vocal nuclei in vocal learners, there was increased expression in ASt, AN, and AMV without the presence of negative expression regions of vocal nuclei (**Fig. 13A** **b, 13B**). The expression was more diffuse as seen in female zebra finches. In the posterior cerebrum, there was increased expression in the DLN region lateral to where we would expect to find songbird HVC, and in the LAI region lateral to where we would expect to find songbird RA (**Fig. 13A** **b, 13B**); in ring doves, the location of the arcopallium relative to the rest of the cerebrum is intermediate in medial-lateral position between that of songbirds and parrots. Finally, there was higher expression in the PLN and adjacent PLMV dorsolateral to where we would expect to find songbird NIf and Av, but the relative increases were not as large due to the unusually high basal levels of ZENK expression in these and the adjacent auditory pathway areas of sitting hearing intact animals (**Fig. 13A** **b, 13B**). Nevertheless, the increase was independent of auditory and visual input, since the birds were deafened and in darkness. In the olfactory bulb, there was no significant difference between sitting still and walking animals (p>0.05, one-tailed t-test).

Summarizing part III of this study, vocal non-learners appear to have movement-associated gene expression in brain areas similar to that of vocal learners. The expression patterns in the cerebellum and AH and AMD somatosensory areas are quite similar across the vocal learning and vocal non-learning groups. The expression patterns in vocal non-learners where one would expect to find vocal nuclei are more uniform without the presence of negative regions due to vocal nuclei.

## Discussion

This is the first study we are aware of to globally map movement-associated areas in the avian cerebrum. The discovered areas are adjacent to the cerebral vocal nuclei in all three vocal learning orders. The anatomical extent of the movement-associated areas are larger than the vocal nuclei, which is consistent with a greater amount of musculature involved in the control of limb and body movements relative to that for the syrinx. Below we discuss the implications of our results for understanding motor and somatosensory pathways in birds, and the evolution of brain pathways for vocal learning.

### Movement-associated brain areas in birds

It is well established that voluntary movements in mammals are controlled by motor and somatosensory pathways in the cerebrum. Motor cortical areas send commands to lower motor neurons in the brainstem and spinal cord that control muscle contraction and relaxation, and to motor basal ganglia areas that modulate ongoing movements, whereas muscle spindles send proprioceptive feedback to somatosensory areas that sense and modulate ongoing movements [56]. However, the motor and somatosensory pathways function in an overlapping manner: the somatosensory cortex sends efference copies of sensory commands to motor cortex prior to the motor command, and the motor cortex has inhibitory connections with the somatosensory cortex to modulate the somatosensory input [57]. Although cerebral motor pathways are well understood in the mammalian brain, surprisingly little is known for the avian brain. Here we found that limb and body movements result in activation within specific cerebral areas of the two known somatosensory pathways (AH and AMD; Nb and MVb). There was striking specificity in the activation patterns when other sensory factors (vision and audition) were eliminated, allowing us to map functional domains of the avian cerebrum. Although such specificity cannot be readily revealed by tracer studies, such studies have shown that the AH area of zebra finches, owls, and pigeons receives input from AMD and from the anterior portion of the intercalated lamina of the hyperpallium (IH), which in turn receives input from the somatosensory thalamus and spinal cord dorsal column nuclei, which are innervated by somatosensory neurons from the wings and legs [58]–[61]. AH also sends descending projections to the intermediate grey matter of the spinal cord [59]. Some have interpreted this pyramidal tract-like projection to the spinal cord to indicate that AH may also have motor or mixed somatosensory-motor functions, and is the homolog of the mammalian motor cortex [62]. Wild and Williams [59] who discovered this projection proposed instead that it is not motor, but somatosensory feedback to the ascending somatosensory pathway of the spinal cord. As for the other pathway, B also receives somatosensory input and projects to Nb [63] and Nb projects to MVb [64]; it is not clear where MVb projects to. Electrophysiology studies show that B has a somatotopic map of the body in budgerigars [65], whereas AH has a touch somatotopic map of the leg and foot in owls [60]. Presumably the areas that sense leg movements during hopping or wing movements during whirring were specifically activated in our study. This activation could conceivably be used for processing proprioceptive feedback from muscles spindles and/or skin touch receptors as the animal sense the floor with its feet or surrounding air with its wings, an idea that can be tested with peripheral stimulation and removal of somatosensory input.

There was also consistent cerebellum activation, as would be expected because the cerebellum receives somatosensory input and sends motor output commands for fine coordination of movements [53], [66], [67]. Our findings are the first that we are aware to identify patterns of movement-associated IEG activation in the cerebellum. The cerebellum in birds, as in mammals, has two somatotopic body representations: one in the anterior half from lobules I-VI and the other in posterior lobules IX-X [67]; determining more specific topographic organization has yielded conflicting results [53]. Connectivity data in pigeons and zebra finches suggest overlapping zones where lobules I-III and IXab receive input from the neck, III-V from the wings, III-VI and IXcd from the legs, VII-VIII from visual and auditory areas but also from somatosensory AH [66], [67]. Our findings are partly consistent with this picture, in that wing whirring activated IEG expression mostly in lobules II-VI, hopping in lobules VI and IXcd, and when moving in dim light or in the dark little if any activation occurred in VII-VIII. In general, the cerebellum activation patterns suggest that limb movements may be mostly responsible for the overall brain gene activation seen in the controlled hopping movement groups, as lobules II-VI and IXcd that are connected to the limbs were consistently activated.

With known somatosensory areas identified, a remaining question is where are the motor areas? Besides the hypothesis that AH and AMD are motor in addition to somatosensory [62], previous studies have suggested the arcopallium and dorsal striatum as general motor areas of the avian cerebrum [68], [69]. However, none that we are aware of have used movement behavior to map a cerebral motor system. Based on our results, we hypothesize that a general motor system in birds consists of the brain areas adjacent to the cerebral vocal nuclei of vocal learners. Our reasons are as follows: First, we found a close association in locations and size of these movement-associated areas with the cerebral vocal nuclei. Second, like the vocal nuclei [32]–[34], these brain areas have movement-associated IEG expression that is independent of auditory and visual input. Third, like the vocal nuclei, the expression levels correlate with the amount of movement performed. Fourth it appears that the vocal nuclei and adjacent regions have similar connectivity, as described below.

**Figure 14:**
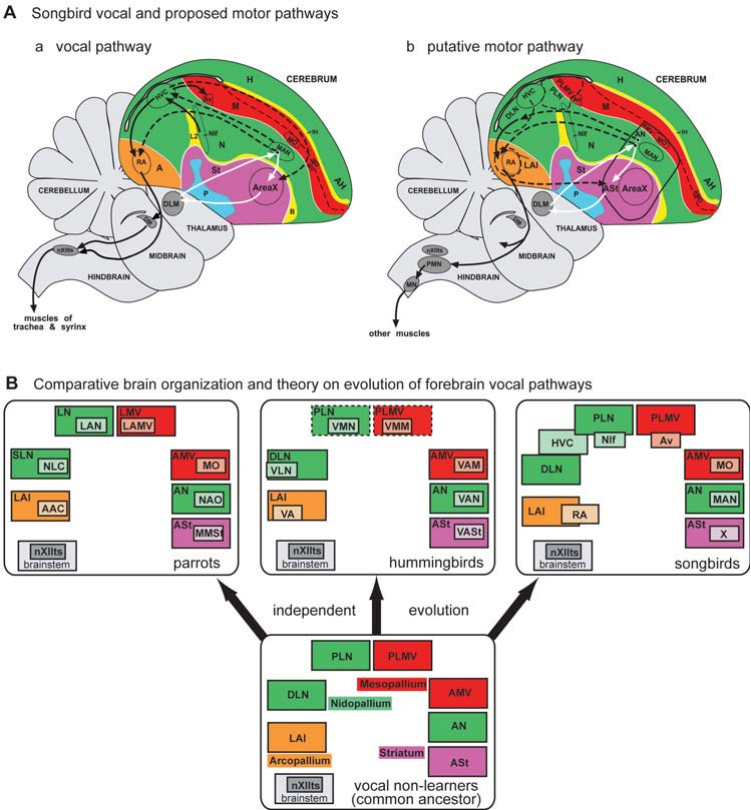
Summary of the results of this study and proposed theory. A. Schematic drawing of the known vocal pathway in songbirds (a) and the putative adjacent non-vocal motor pathway in all birds (b). Movement-associated areas adjacent to the posterior vocal nuclei (HVC, RA, NIf, and Av) in (b) are drawn with dashed lines to indicate that they are lateral to the plane of the section shown. Lines and arrows in (b) are inferred from our compilation of the literature on tracers placed adjacent to the vocal nuclei (Table 3). White arrows: connectivity of anterior vocal pathway (a) and proposed adjacent anterior motor pathway (b). Black arrows: connectivity of posterior vocal pathway (a) and proposed adjacent posterior motor pathway (b). Not all known connections are shown; in particular, the anterior mesopallium connections have not been determined in songbirds, a DLN to ASt connection appears to be weak in zebra finches, and RA and LAI also projects to other sub-telencephalic areas (Table 3 and references therein), and connectivity of PLN and PLMV with other movement-associated regions is not known. Different background colors designate different cerebral brain subdivisions. B. Diagram comparing brain organization in the three vocal learning groups and in a vocal non-learner as a proposed common ancestor. We hypothesize that by independent evolution, the vocal nuclei (light colored boxes) of recent vocal learners originated from the movement-associated brain areas (dark colored boxes) of the common ancestor. Relative sizes and positions of brain areas are approximate. The parrot posterior regions (LAI, SLN, LAN, and LAMV) are more anterior and laterally than the corresponding areas in the other species. Hummingbird PLN and PLMV are highlighted with dashed lines to indicate that they were only examined in a few birds. Songbird HVC is drawn as adjacent to both DLN and PLN, and thus, it is ambiguous as to which region it could have evolved from. Color-coding in panel (B) reflects the coding of panel (A).

Many prior studies have accidentally or purposely placed tracers adjacent to the songbird vocal nuclei, and except for the HVC shelf and RA cup [23] the function of these brain regions were not known. In some of these studies on zebra finches, we note remarkable overlap in the connectivity patterns [70], [71] with the movement-associated gene expression patterns (this study). When we compile these connectivity results with the movement-associated gene expression results (**Table 3** [63], [70]–[74]), it appears that the movement-associated areas in songbirds may be connected in anterior and posterior pathways in parallel, although not identical, with the adjacent vocal nuclei (**Fig. 14A**). The anterior movement-associated areas, like the anterior vocal pathway nuclei, appear to be connected in a pallial-basal-ganglia-thalamic-pallial loop (**Fig. 14A** **;** white arrows): anterior AMV to AN adjacent to LMAN, these two areas to the striatum adjacent to Area X, the striatum via its pallidal-like neurons to the dorsal thalamus adjacent to the vocal part of DLM, and the dorsal thalamus back to the AN adjacent to LMAN. Connectivity of MO or the surrounding MV is not known in songbirds, but the comparable song nucleus and adjacent MV in parrots and the MV in pigeons projects to the anterior nidopallial and striatal vocal nuclei and surrounding area, respectively [75], [76]. The parrot anterior vocal pathway also forms a pallial-basal-ganglia-thalamic-pallial loop (**Fig. 1B**) [76]. That is, similar connectivity can be compiled for these cerebral regions in other vocal learning and vocal non-learning birds [25].

**Table 3:** Sources for connectivity of neural populations adjacent to songbird vocal nuclei

Connection	Direction	References
Ast = >DT	anterograde	[39], [71], [74]
DT = >AN	retrograde, anterograde	[70], [72]
AN = >LAI & ASt	retrograde, anterograde	[63], [70]–[72]
AN = >DLN	retrograde, anterograde	[70], [71]
DLN = >ASt	retrograde (weak in finch, strong in pigeon)	[71], [75]
DLN = >LAI	retrograde, anterograde	[63], [71]
LAI = >PMN	retrograde	[63], [71]

The first column shows the connection; arrows indicate axonal projections. The second column indicates the tracer direction studied; connections determined in both the anterograde and retrograde directions are accepted with more confidence than ones determined only in one direction. The third column lists the references where these connections were determined. The AN = >LAI & ASt indicates that AN neurons send collateral projections to LAI and ASt adjacent to RA and Area X respectively, which is similar to the collateral projections from individual LMAN neurons to RA and Area X. In the studies before 2004, the old avian brain nomenclature was used and terminology varied between studies. AN was called the LMAN shell or frontal neostriatum, DLN we consider here as a part of NCL (see main text), LAI was called dorsal archistriatum (Ad), and ASt and Area X was within a region called the lobus parolfactorius. PMN is premotor neurons of the brainstem, medulla, and possibly spinal cord. Here, we adopted a terminology that is applicable across all avian species, vocal learners and non-learners.

The posterior movement-associated areas in zebra finches, like the songbird posterior vocal pathway, appear to be connected into a descending motor system: the DLN posterior and lateral to HVC (within a larger region called the caudal lateral nidopallium, NCL) [22], [63], [70], [71], [73] projects to LAI directly lateral to RA, which in turn projects to pre-motor neurons (PMN) of the brainstem reticular formation (**Fig. 14A**). Interestingly, the reticular PMN in pigeons, chickens, and ducks also receive a direct projection from the arcopallium; in these species, the reticular PMN laterally adjacent to the nXIIts vocal nucleus projects onto the spinal cord motor neurons that control muscles for wing and leg movements, and when stimulated electrically or with neurotransmitters, induce wing beats, hopping, or walking [77], [78]. The parrot and hummingbird posterior vocal nuclei also make a similar descending motor projection (**Fig. 1B**) [30], [76], [79].

In terms of apparent connectivity between posterior and anterior movement-associated areas, the shell of neurons around songbird MMAN and the comparable area in non-songbirds projects to NCL (inclusive of DLN) in a similar manner as MMAN projects to HVC [71], [80]; the shell around LMAN projects to LAI in a similar manner as LMAN projects to RA (**Fig. 14A**) [70]. Differences within the vocal pathway are as follows: unlike HVC's projection to Area X, the adjacent DLN in zebra finches only sends a weak projection to the striatum, whereas the LAI adjacent to RA sends a strong projection to the striatum (**Fig. 14A**) and many other areas besides the reticular PMN [71]. Likewise, there are more differences in the connectivity between the posterior and anterior vocal pathways of songbirds and parrots than there is within each of the vocal pathways (**Fig. 1B**) [10]; for example, in both groups, output of the anterior vocal pathway to the posterior vocal pathway is via the MAN-like nucleus, but the input is either from the HVC-like nucleus (songbirds) or the RA-like nucleus (parrots).

Interestingly, in zebra finches, AH sends some of its heaviest cerebral projections to the areas around the anterior vocal pathway nuclei, to the DLN lateral to HVC, and to ventral AI [81]; this connectivity pattern is also strikingly similar to the movement associated gene expression we found (**Fig. 14A** **b**). This overlap of connectivity and gene expression patterns suggests that AH may transmit somatosensory input into putative anterior and posterior motor pathways adjacent to vocal nuclei, or that the areas adjacent to vocal nuclei are somatosensory instead of motor. If the latter possibility were true, however, there would be no activated cerebral areas left for the motor control of movement. As for the vocal nuclei themselves, they do not require somatosensory input from the syrinx for their vocalizing-driven gene expression and this is one reason why this gene expression has been designated motor-driven [33]; the vocal nuclei also show pre-motor neural firing during singing [32], [34]. Thus, based on parallels with the vocal nuclei, we hypothesize that the movement-associated areas adjacent to the vocal nuclei will show both pre-motor firing for movement control and somatosensory feedback firing from AH after initiation of movement. These hypotheses on connectivity and activity can be confirmed or falsified in future studies that perform double labeling experiments with injected neural tracers and movement-induced IEG expression, that locally remove cerebral somatosensory input into posterior and anterior movement-associated areas, and that perform electrophysiological recordings during movement. Preliminary electrophysiological studies from our group indicate that the AN area adjacent to zebra finch LMAN has pre-motor neural firing during hopping (Tremere, Pinaud, and Jarvis; Soc. Neurosci. Abstracts, 2007, 221.9).

Although future work still needs to be conducted to decipher motor and/or somatosensory roles of the identified brain areas of this study, our findings help advance future investigations of the avian brain. Since it is difficult to make birds sit absolutely still during a sensory task and obviously impossible during behavioral tasks, general movement-associated activation may distract experimenters' attention from other types of activation. Thus, knowing the brain areas activated during movement will be important for studies using behavioral molecular mapping to identify brain areas involved in specific behaviors, including sensory-motor learning tasks.

Taken together, we speculate that the vocal control circuits, which use auditory information to influence vocal motor output are anatomically adjacent to putative motor control circuits, which use somatosensory information to influence motor output. We suggest that the motor control circuits comprise a general cerebral motor system consisting of two sub-pathways: an anterior and a posterior pathway (**Fig. 14A** **b**). Based on parallels with the vocal system, we hypothesize that the adjacent posterior pathway controls the production of movements and the adjacent anterior pathway controls sequencing if not learning of movements. Although, our results do not indicate whether the anterior areas are involved in motor learning (such as learning to walk, fly, or manipulate the beak), as is the case for the anterior vocal nuclei during song learning, like the anterior vocal pathway's activation during singing, the adjacent movement-associated areas are active during production of motor behaviors. Our approach was not specific enough and did not aim to map a possible homunculus organization as seen in the mammalian motor cortex [82], but given that the avian AH and AMD has somatotopic organization [60], [65] and that restricted patterns of activation occurred adjacent to the vocal nuclei depending on the types of movements performed, it would not be surprising to find a homunculus-like organization in a putative avian anterior and posterior motor system as well.

### A motor theory for vocal learning origin

Based on the above findings and related studies, we propose the following theory: *Cerebral systems that control vocal learning in distantly related animals evolved as specializations of a pre-existing motor system inherited from their common ancestor that controls movement, and perhaps motor learning*.

Although the results of this study do not prove this theory-the reason for calling it a theory-they support it more than other theories of vocal learning origin; we called our idea a theory as opposed to a hypothesis, because it consists of multiple hypotheses. Others have suggested that forebrain vocal learning systems for learning and production in various species including humans have evolved out of either a pre-existing auditory pathway [22], [23], an auditory-motor system [83], a non-motor cognitive system [84], [85], or de-novo [86]. Support for some of these theories in birds are: i) The songbird posterior vocal nuclei are adjacent to and share similar connectivity with the descending auditory pathway [23], [87]; ii) The parrot posterior vocal nucleus NLC is surrounded by auditory responsive neurons [88]; iii) All vocal nuclei of songbirds show neural firing when hearing song, leading to the motor theory of song perception [89]; and iv) The striatum around Area X was thought to show hearing-induced IEG expression [46], [47]. Alternative explanations or interpretations can now be offered for these findings. These include that: i) The descending auditory pathway, which is not activated by movement, has connectivity that is similar to movement-associated areas [23] (noted here) and thus this may not be a good distinguishing factor; ii) The auditory-evoked neural firing in the songbird vocal nuclei occurs mainly in anesthetized or sleeping birds [90], [91] whereas when the birds are awake, firing and IEG induction is mainly motor-driven (singing) independent of auditory input [33], [34]; iii) Similar to auditory responses in vocal nuclei of anesthetized songbirds and parrots, the auditory responses adjacent to NLC in parrots [88] may reflect sensory input into a motor system, in that parrot SLN around NLC behaves more like DLN adjacent to songbird HVC than to the auditory shelf adjacent to HVC; and iv) The hearing-associated gene activation around anterior songbird vocal nuclei can be explained by animals moving in response to hearing song (this study).

These alternative explanations and interpretations do not mean that auditory or other sensory information does not enter the vocal or putative adjacent motor system. On the contrary, sensory information must enter the systems in order to control sensory-motor guided behavior. For auditory input, the best candidate songbird nuclei so far are the vocal nucleus NIf [92], [93] and the auditory CM (MV-L2) area [21] in which Av is located. Interestingly, the adjacent PLN and PLMV regions were the only two areas that showed both movement- and auditory-associated activation independent of each other, and thus we speculate that they could possibly represent a pre-existing multimodal brain cluster where auditory information is transmitted to a motor system. Likewise, one view for budgerigars is that the vocal nucleus proposed to be analogous to NIf (LAN [6]) receives input from auditory fields L1 and L3 [76], [94]; but this remains to be further tested and considered with possible dual auditory input from an auditory part of basorostralis in parrots [30], [95].

Our theory can explain why the cerebral vocal systems are similar across distantly related vocal learning birds (**Fig. 14B**). For many years, there has not been a satisfactory explanation for the finding that songbirds, parrots, and hummingbirds have seven comparable cerebral vocal nuclei that cannot be found in their close vocal non-learning relatives [7], [13]–[15], [96]. One possible explanation was that supposed vocal non-learners actually have rudimentary vocal learning behavior and rudimentary cerebral vocal nuclei that were then independently amplified in vocal learners (**Fig. 1A**); however, none have been found despite efforts to search for them [13], [15], [79] (and this study). Another explanation was that a cerebral vocal pathway existed in a common vocal learning ancestor of vocal learning birds that was then lost multiple independent times in their cousins [7] (**Fig. 1A**, green dots). A third and the dominant hypothesis was that each of the three vocal learning bird groups evolved similar cerebral nuclei for vocal learning and production independent of their common ancestor [7], [14] (**Fig. 1A**, red dots). Our theory offers a modified view of the independent evolution hypothesis, this being that the three vocal learning bird groups independently evolved similar cerebral vocal systems but that were dependent, i.e. constrained, by a previous genetically determined motor system inherited from their common ancestor (**Fig. 14B**). This pre-existing motor system may be a basic motor system of the avian brain that consists of distinct areas (possibly seven nuclei) distributed into two pathways (posterior and anterior), which in parallel incorporate portions of different cerebral subdivisions (mesopallium, nidopallium, arcopallium, and striatum), each sub-serving a specific function. If true, then such a basic posterior/anterior motor system that controls different non-vocal muscles in parallel pathways via premotor neurons in the brainstem could be used as a template for the evolution of a vocal motor/learning system that controls muscles of the syrinx, taking over control of DM and nXIIts that normally controls innate vocalizations. This hypothesis may be testable with fate mapping and genetic manipulation studies of developing brain circuits.

One potential caveat of our theory is that the posterior vocal nuclei are in different locations in each vocal learning avian order and so are the adjacent movement-associated regions. The biggest relative differences are seen in parrots compared to all the other species (songbirds, hummingbirds, ring doves, chickens, quails, pigeons, and suboscines) we have examined in our studies. One possible explanation is that the posterior motor pathway migrated more anterior-laterally during evolution of the parrot ancestor and that the posterior vocal pathway moved with it or later evolved out of it. Support for this general idea is that the arcopallium in parrots is positioned much further anterior relative to other avian species, although it is still posterior relative to the anterior vocal nuclei. If the motor part of the nidopallium moved with the arcopallium anterior and laterally, then this would suggest that parrot SLN is the homolog of DLN in the other species (**Fig. 14B**). Further, the parrot cerebrum, and the nidopallium in particular, is much larger in brain to body size ratio relative to other species [97], [98]. Since the posterior nidopallium also contains sensory integration pathways [99], perhaps such sensory pathways were expanded in parrots displacing the posterior motor pathway anterior and laterally. This idea can be tested by mapping the functional organization of the nidopallium and the connecting arcopallium between the auditory and posterior movement-associated areas in parrots relative to other species. In hummingbirds, the posterior movement-associated areas are in a more similar position relative to songbirds and ring doves, but the posterior vocal nuclei are positioned more lateral instead of medial to the movement areas. Such differences suggest that it is likely that the vocal nuclei in each bird order evolved independently, but from the common ancestor motor pathway substrate (**Fig. 14B**).

We believe that our findings may also have implications for understanding the evolution of brain pathways for vocal learning among distantly related mammals. The phylogenetic distances among vocal learning mammals (humans, bats, sea mammals, and elephants) are similar to those among vocal learning birds [10]. Comparative analyses among vocal learning and non-learning mammals [10], [100], [101] and between mammals and birds [10], [102], with humans being the only vocal learner for which cerebral vocal (speech) brain regions are known, indicate some analogies between humans and vocal learning birds [10]. These include in humans a proposed anterior vocal pathway involving Broca's area, adjacent cortices, the anterior striatum, and anterior thalamus and a posterior vocal pathway that comprises the face motor cortex and its projections to brainstem vocal motor neurons [10]. The face motor cortex is within the motor cortex and Broca's area is adjacent to or considered by some to be within the pre-motor cortex [103]. An analogous area of the pre-motor cortex in non-human primates, macaques, a vocal non-learner, modulates orofacial, but not laryngeal, movements [104]. Further, the mammalian non-vocal motor (posterior) and pre-motor (anterior) pathways follow a connectivity design similar to the songbird and parrot posterior and anterior vocal pathways [10], [76]; these are the mammalian descending motor pathway and cortical-basal-ganglia-thalamic motor loops, respectively. Perhaps the evolution of vocal learning brain areas for birds and humans exploited a more universal motor system that predates the split from the common ancestor of birds and mammals, i.e. stem amniotes [31]. Such a universal system would be consistent with both proposed hypotheses of avian and mammalian pallial homologies, which are that pallial areas containing the vocal nuclei in birds are homologous to either the mammalian six layered cortex or to the mammalian claustrum-amygdala complex [31], if the latter in mammals were found to consist of a rudimentary motor system. This hypothesis can be strengthened or weakened by studying brain pathways for vocal learning in other vocal learning mammals as well as non-vocal motor pathways of reptiles and amphibians.

At this point, we cannot say in our theory whether the forebrain vocal system formed by using a pre-existing part of a motor pathway as a scaffold or usurped a pre-existing part of the pathway. However, we do not believe that a pre-existing part of a motor pathway was lost. Rather, our theory is in line with previous ideas on evolution of novel brain systems from older systems. For example, Finlay [105] suggested that new mammalian cortex areas arise first by an enlargement of an older region and then second by allocating part of that older region to the new function, while the remaining part maintains the old function. This is similar to the idea that new functions can be generated by gene duplications, where a gene is duplicated and one copy is used for a new function while the old copy maintains its function [106]. More universally, Ghysen [107] argues that vertebrate as well as insect brains have ancient principle sensory and motor circuits with stable functions upon which alterations by gene mutations and embryonic development during evolution are applied to home new functions. These altered circuits may then be uncoupled from the original pathways to allow the novel functions without affecting the original system. Perhaps vocal learning systems have evolved by such a mechanism.

Although our findings led us to propose the above theory, we are not the first to implicate a motor origin for a learned vocal behavior. Based upon a literature summary of studies conducted in humans, Robin Allot in a linguistic conference proceedings [24] proposed a “motor theory for language origin” where he argued that language brain areas evolved from a pre-existing motor neural system; however, he did not provide experimental evidence or flesh out the anatomical or mechanistic details of this theory. Lieberman [100] proposed that language areas evolved out of a pre-existing cortical-basal-ganglia-thalamic-loop, for which he deemed the basal ganglia part as the reptilian brain. However, we now know that reptilian and avian cerebrums are not made up of only basal ganglia, that vocal learning birds only have part of the vocal system in the basal ganglia, and that spoken language areas may involve more than just this loop [10], [31]. Farries [73] and Perkel [102] proposed in birds and Jarvis [10] in birds and humans, that vocal learning pathways in birds and humans may be similar to systems outside of the vocal pathways that intuitively could be motor pathways found in vocal non-learning birds and mammals; but they did not have experimental evidence to corroborate these suggestions. Here we provide evidence that the brain areas adjacent to the vocal systems of all known vocal learning birds function during movement. This poses the question-what makes vocal learning, and spoken language for that matter, special-a question that is often debated [83], [108]-[110]. We argue that it is a cerebral motor system that controls the vocal apparatus. That is, vocal learners and non-learners have similar auditory pathways, but vocal learners have a unique vocal motor system that gives them the ability to translate auditory signals into vocal signals. Like in birds, it is not clear how the auditory information reaches the vocal motor areas but a dorsal sensorimotor stream from secondary auditory cortex to Broca's area has been one hypothesized system [111].

Our results are also concordant with the gestural origin of spoken language hypothesis, where the motor learning ability of gestures in humans and non-human primates has been argued to be the precursor behavior for motor learning of speech/language [112]–[114]. During child development, gesture production appears before speech production and is thought to enhance learning of speech; adults also use limb gestures automatically and often unconsciously during speech production [112], [115]. This gesturing hypothesis was one basis for the motor theory of language origin [24]. We suggest that, logically, gesturing is controlled by a pre-existing motor system. Gesturing, although not a requirement in our theory, has not been well studied in birds, but many avian species perform other movements such as a courtship dance or wing displays during vocalizing [116]–[120]. Investigations into the behaviors and neural circuits for movement displays in birds may help shed light onto these ideas. If verified in both birds and mammals, then the evolution of vocal learning brain systems as a specialization of a pre-existing motor system could be a general feature of the vertebrate brain.

## Materials and Methods

### Species

We used 38 garden warblers (28 males and 10 females), 35 zebra finches (31 males and 4 females), 9 budgerigars (both males and females have vocal nuclei), 6 male ring doves, and 9 male Anna's hummingbirds. Many of these animals also provided data for multiple prior studies [6], [26], [27], [37], [42], [121]–[125], thus leading to a large gain of knowledge from a relatively small number of animals per published study. The garden warblers were caught on Helgoland and around Oldenburg (Germany) in May-September, 2002/2003, and acclimated to captivity for a minimum of 5 days. Zebra finches and budgerigars were obtained from either local breeders or our breeding colonies at The Duke University Medical Center (USA). Ring doves were obtained from Dr. Wilmer Miller at Iowa State University (USA). Anna's hummingbirds were obtained with the help of Dr. Douglas Altshuler at the University of California, Riverside (USA). All animal procedures were approved by the Institutional Animal Care and Use Committee of Duke University, or of Bezirksregierung Weser-Ems (Oldenburg) for garden warbler experiments, or University of California Riverside for hummingbird experiments.

### Behaviors

For all species, we placed birds inside an experimental apparatus for at least 1–3h before the start of an experiment and carefully observed the birds' behavior from at least 1h before until the end of the experiment. The 1–3h waiting period allowed for the decay of any gene expression induced either by behaviors before the observation period and/or by the light-dark transition [33]. We only collected a bird after it produced repetitive movement behavior or was sitting still but awake while a minimum of other behaviors occurred for at least 30–45 min, the peak time of ZENK mRNA expression [33], [45]. To reduce hearing-induced gene expression [33], [46] due to noise, we made sure that no sounds from the experimenters or from the outside reached the birds during the critical hour. In case of accidental noise (from indoors or outdoors), we waited at least 1h before starting the experiment again. When the required highly consistent behavior was observed for 30–45 min, we sacrificed the bird, rapidly dissected its brain within 5–6 min, separated the two hemispheres along the mid-sagittal plane (except for hummingbirds), embedded them in TissueTek O.C.T. (Sakura Finetek, NL), and quick-froze the brains to −80°C in a dry ice/ethanol bath.

#### Migratory restlessness: wing whirring and flights

To reliably record and quantify migratory restlessness movement behavior in garden warblers and relate this behavior to activity-dependent gene regulation, we designed a behavior apparatus as described in **Figure S1A** [26], [37]. This apparatus allowed us to carefully observe the bird's behavior in real time. We found that garden warblers under dim light either sit in this orientation cage for extended periods of time or perform migratory restlessness behavior more consistently and stereotypically than they do in the Emlen funnels [35] normally used for orientation experiments. This behavior included head scans apparently to detect the direction of the Earth's magnetic field [37], some hopping, but mainly rapid wing whirring while perched (92%±1.34% SEM of movement events, n = 15 birds).

On the day of testing, a thin stripe of infrared-retroreflective tape (3M) was glued to the top of the bird's head. The tape was used to track and record the bird's movements with an infrared-sensitive video camera under dim light. For the dim light conditions, the birds were placed in the cylindrical cage before 13:30h and food was removed 90 min before onset of darkness (simulated local photoperiod). Some birds were exposed to an artificially changed magnetic field, but these manipulations did not affect the IEG expression in movement-associated areas, as described elsewhere [27]. We collected garden warblers in four groups: 1) animals that sat relatively still in the cage during the day time showing a minimum of movement behavior (e.g. less than 5 flights, 100 wing beats, and/or 50 head scans in 1h, n = 5); 2) animals that remained awake and still during the night in dim light (0.04 lux, n = 11); 3) animals that displayed general motor activity, mostly flights, during the day (e.g. several hundred to several thousand defined movement events; n = 5); and 4) animals that displayed wing whirring behavior in dim light (n = 15). We also placed zebra finches in the cylindrical cage and collected animals under conditions of groups 2 (n = 4) and 3 (n = 4) above.

To quantify the **number of wing beats**, we measured both the rapid wing whirring made during migratory restlessness behavior in dim light and wing flapping made during day light conditions often in preparation to fly off the perch. The amount of wing beats during flapping was relatively simple to quantify manually from video, as the birds performed it at a slow rate. Quantifying beats during wing whirring was more complicated, as it was rapid. To measure wing beats during whirring, we first measured the mean wing beat frequency for several birds by watching the video frame-by-frame. This mean frequency (average 11 wing-beats/sec) was then transferred to the keys of a PC keyboard so when a key assigned to a Matlab program was pressed continuously, the output signal was identical to the wing-beat frequency. Thereafter, the observer watched all video tapes in real-time and either held the key down for the time the bird performed constant wing-whirring or flying in the cage or made individual key-strokes for isolated wing flaps while perched. The time and number of each button-press event were analyzed using a custom-written Matlab program. To quantify the **number of flights**, the same videos were reviewed and we counted the number of times the bird flew off the perch or off the bottom of the cage. When zebra finches were placed in the cylindrical apparatus during the day light, they spontaneously hopped around the perimeter of the cage as well as performed some flights; in dim light, they remained still. We quantified the movement behaviors in day light, but there was not enough variation among animals to perform correlations.

#### Hopping and Walking

To reliably induce repetitive hopping or walking behavior in birds, we designed a behavior apparatus consisting of a cylindrical, transparent plexiglass wheel, placed inside a sound isolation chamber, attached to a metal rod that was controlled by a relatively quiet motor outside of the box with variable speed control (**Fig. S1B**). The inner floor-surface of the wheel was covered with a black rubber mat to give traction for their feet. Behavior was observed and recorded via an infrared-sensitive camera inside the sound-proofed box, connected to an external video recorder. In dim light (∼0.04 lux) or dark (in the box, which was in turn inside a room without windows or light), most birds sat still.

To perform an experiment, we first placed a bird inside the apparatus during normal waking hours and rotated the wheel (∼20 rpm) in day light for 5 min and then in dim light or darkness for an additional 10 min to get the bird accustomed to the wheel and reduce stress in the new environment; both species tested in the wheel (zebra finches and budgerigars) habituated fairly easy to this task. We then turned off the wheel and allowed the bird to sit for 2-3h in dim light or darkness; because this was in the middle of the day, most birds did not go to sleep as determined by eyes open and head not resting on the back. Thereafter we collected 8 groups of zebra finches after 30 min of: 1) males that remained awake and sat still in dim light (n = 3); 2) males that sat still in the dark (n = 3); 3) males that hopped in the rotating wheel in dim light (n = 3); 4) males that hopped in the rotating wheel in the dark (n = 3); 5) deafened males that hopped in the rotating wheel in the dark (n = 3); 6) males that remained still in the dark outside the wheel while hearing another bird hop inside the wheel (n = 2); 7) males that sat still in the dark hearing playbacks of conspecific songs (three different songs spaced every 10 seconds per min for 30 min; n = 3); and 8) deafened females that hopped in the rotating wheel in the dark (n = 4).

For budgerigars, we collected three groups: 1) animals that remained awake and sat still in the wheel in dim light (n = 3); 2) animals that hopped in the rotating wheel in dim light for at least 30 min (n = 3); and 3) deafened animals that hopped in the rotating wheel in the dark (n = 3).

For ring doves, we used a treadmill (37×14.5×30 cm; Simplex II, Columbus Instruments, Columbus, OH) designed for rats and loaned to us by Dr. Miguel Nicolelis (Duke University). The treadmill was attached to a 4-meter cable controlled by a relatively quiet variable speed motor, which was placed in an adjacent room. An infrared video camera was used to observe the animal's behaviors. The doves were prepared in a similar manner as above for the other species. After the 2–3h quiet period, we collected two groups of birds: 1) males that remained awake and sat still in the dark for at least 30 min (n = 3); and 2) deafened males that walked on the treadmill (∼10 rpm along the 37 cm length) in the dark for at least 30 min (n = 3).

#### Hummingbird Flying/Hovering

To reduce auditory-induced and visually-induced gene expression in Anna's hummingbirds when they fly, the feathers around one ear were trimmed (sides randomly alternated among birds), an ear-plug of clay placed gently in the ear canal, and then the ear and eye of the same side was covered with three layers of black vinyl electrical tape; one layer faced inward so that the smooth surface covered the eye to prevent damage to it. The tape was then sealed at the edges with super glue to the surrounding skin and feathers to reduce light leakage. Left and right sides were alternated to control for any possible lateralization differences. Thereafter, the bird was placed inside a transparent square plexiglass box we designed (20×30.5×20 cm) with a wooden perch ∼12 cm above the floor across the center of the box, inside a room with dim light. After making some attempts to escape, most birds within minutes learned the location of the plexiglass barrier and then rested on the perch. After 1–1.5h of sitting on the perch, some birds began to spontaneously fly and hover in a repetitive fashion (hummingbirds have a relatively unique flying behavior of being able to hover). This behavior included a hovering lift off the perch, a small circular flight trajectory (∼10–15 cm in diameter) above the perch in the direction of the opened eye, and then landing on the perch. A video camera was used to record the behavior. Using this paradigm, we collected two groups of Anna's hummingbirds: 1) unilateral ear and eye covered males that remained awake and sat relatively still (less than 20 movements) on the perch for at least 30 min (n = 3); and 2) unilateral ear and eye covered males that made at least 60 or more circular hovering flights in 30 min (n = 4).

#### Singing

To compare movement-associated gene expression with singing-driven gene expression in vocal learning birds, we chose previously collected brain sections of zebra finches (n = 6) and budgerigars (n = 6) [6], [125] that sang alone or heard song with a minimum of other movement behaviors, or brains of Anna's Hummingbirds (n = 2) that sang near a feeder in the open field following a previously described protocol used for other hummingbird species [7]. We used animals that sang 60 or more song bouts of undirected song within a 30 min period; undirected singing leads to high IEG expression in all known vocal nuclei [5].

#### Hearing and Deafening

The “hearing song”, “hearing a bird hop”, and “hopping deaf” groups are mentioned above in the hopping and walking experiments. We further describe the procedures for these groups here. To assure that birds remained still, those that heard the hopping bird and rotating wheel were gently wrapped in cotton cloth bedding, where they remained for the ∼2h habituation period and throughout the stimulus period. For experiments that required elimination of auditory input, birds were deafened by bilateral cochlea removal following a previously described protocol [126]. Briefly, birds were anesthetized, a small hole was cut in the neck muscle and the skull behind the ear, the oval window of the cochlea removed and then the cochlea removed. The skull and skin were sealed with tissue adhesive, and the bird allowed to recover for 4–6 days. Thereafter, the bird was placed inside of the wheel (zebra finches or budgerigars) or treadmill (ring doves) and treated the same as all other groups.

### Gene expression analyses

For each brain, 12 µm frozen sections were cut throughout the entire left hemisphere in the sagittal plane. We used sagittal sections to maximize the amount of brain tissue per section. For selected example birds, serial coronal sections were also cut throughout the entire right hemisphere. Corresponding sections of all birds of a given experiment were fixed in 4% paraformaldehyde and processed for in-situ hybridization with antisense ^35^S-UTP labeled riboprobes of zebra finch ZENK (acronym for zif268, Egr-1, NGF-1A, and Krox-24) [125], c-fos [125], GluR1 [15], or FoxP1 (forkhead box P1) [16] cDNAs following previously described procedures [15]. Hybridization temperature and washes were 65°C for zebra finches and garden warblers, 62°C with ZENK and c-fos or 60°C with FoxP1 for all other species. ZENK and c-fos can be detected in neurons ∼10 min after increased neuronal activity with peak expression at 30–45 min [33], [125], and therefore increased cumulative mRNA expression marks brain areas that were active during the last 45–60 min of the animals' life. The hybridized sections were exposed to X-ray film (Biomax MR, Kodak) for 1–4 days, then dipped into autoradiographic emulsion (NTB2, Kodak), incubated for 1–3 weeks at 4°C, processed with Kodak developer (D-19) and fixer, and Nissl-stained with cresyl-violet acetate solution (Sigma). X-ray film brain images were digitally scanned from a dissecting microscope connected to a SPOT-III CCD camera using SPOT imaging software (Diagnostic Instruments, Inc.). Care was taken to use the same lighting settings across all images used for quantifications. We used Adobe Photoshop 7.0 to measure the mean pixel intensities in the brain areas of interest from at least two adjacent sections on a 256 grey scale. For all species, except the garden warblers, we normalized the value of each brain area by dividing it with the value of the entopallium for each animal. This allowed us to compare expression levels across in-situ hybridizations conducted on different days for the large number of experiments performed; the garden warblers were all hybridized at once, and thus did not need normalization.

### Distance and gross volume measurements

To calculate distances, we digitized images from 2–3 sections per animal (n = 3 deaf, dark, hopping animals) containing ZENK expression in movement-associated areas. Images were taken under brightfield and 10X magnification with a Leica DMXRA microscope (2.5X for calculating the MAN to Nb and MO to MVb distances). Nissl stained boundaries were used to locate the vocal nuclei. We measured the distance of the nearest movement-associated areas with the criterion that the region had to have a minimum of five labeled neurons within ∼200 µm radius to prevent artificially identifying non-movement-associated areas with few labeled neurons. We then averaged all values per area per bird to obtain average distance measurements for each bird. To calculate gross relative cerebral volumes of the movement-associated regions and vocal nuclei, we used the digitized X-ray film images of 9 evenly spaced sagittal sections that spanned representative areas of the cerebrum, whether or not the sections had high levels of gene expression. We used Nissl stains of these same sections to locate the vocal nuclei. We then measured the areas of each movement-associated region and each vocal nucleus in each section (0 if not present), added up the values, and divided the value by the total area of the cerebrum across all 9 sections, to obtain a volume fraction for each brain region per bird. Although this method does not give absolute values, it reliably yields relative values. Values were then averaged across birds (n = 3 deaf, dark, hopping animals).

### Statistics

To test for significant differences in IEG expression levels among groups, we performed ANOVAs followed by a Holm-Sidak multi-comparison test when comparing three or more groups of animals (songbird and parrot experiments) and t-tests when comparing just two groups (female zebra finch, hummingbird, and ring dove experiments), using SPSS (Chicago, IL) or SigmaSTAT (Systat Software, San Jose, CA) software. Tests were performed on each brain area separately due to our primary interest in deciphering activation of specific areas across groups and due to the large number of areas, which made multiregional/group comparisons beyond the limits of the software capabilities. To determine correlations between movement behaviors and IEG gene expression levels in garden warblers, we performed two analyses, both using Pearson Correlation. First, since the relationship between movement and gene expression levels was not linear, we searched for a curve that best fitted the data, which was a saturating exponential curve (f = y_0_+a*(1-exp(-b*x))), followed by a Pearson Correlation between the measured values and values deducted from the fitted graph. Second, we performed a double natural logarithmic transformation that resulted in a linear relationship, and then performed a Pearson Correlation between measured values and the regression graph.

### Anatomy

Because the IEG expression patterns form clusters of brain areas across brain subdivisions that can be misleading in defining subdivision boundaries and because we examined multiple species for which cerebral subdivision organization is not well characterized, we sought reliable markers of brain subdivision boundaries to define the anatomical locations of IEG expression. Nissl staining was not suitable for unambiguously defining brain subdivision boundaries. Thus, in addition to Nissl staining, we found that GluR1 [15] and FoxP1 [16] expression patterns were valuable and critical for identifying brain subdivisions in all avian species. GluR1 shows enriched expression in the hippocampus, mesopallium, and striatum, low expression in the pallidum and in primary thalamic recipient neurons (L2, B, part of E, and IH), intermediate expression elsewhere, and differential expression in songbird vocal nuclei (high in AreaX, low in HVC, RA, and MAN). FoxP1 shows highly enriched expression in the mesopallium and striatum, low expression in the hyperpallium and nidopallium, and lower expression in the primary thalamic recipient neurons (L2, B, part of E, and IH), the pallidum and arcopallium. FoxP1 also shows differential expression in songbird vocal nuclei (higher in HVC, RA, and Area X; lower in MAN), the parrot analogs of Area X (higher in MMSt) and HVC (higher in NLC) as previously noted [16], and, as we note here, the hummingbird analog of HVC (higher in VLN).

When using these genes and many others as brain subdivision markers (Jarvis et al, in preparation), it becomes apparent that what has been previously labeled as dorsal hyperstriatum (HD) and ventral hyperstriatum (HV) in the old avian brain nomenclature [29], [31] is marked with mesopallium enriched genes, such as GluR1 and FoxP1. Thus, here we follow the practice of some of our recent publications [26], [27] of labeling the formally named dorsal hyperstriatum (HD) as dorsal mesopallium (MD) and the formally named ventral hyperstriatum (HV) as ventral mesopallium (MV), due to the presence of mesopallium specific gene expression.

For the budgerigar brain we used the term supra-lateral nidopallium (SLN) to describe the area that stretches dorsally, ventrally, and caudally around the vocal nucleus NLC. The caudal and ventral areas have previously been called the superior central nucleus of the lateral nidopallium and ventral nucleus of the lateral nidopallium (NLs and NLv) [30].

In addition to the above definitions, we also sought to define a more global terminology that can be applied across multiple avian species for names of homologous brain structures that are in different topological positions among species. When possible, we used a non-coordinate terminology for this purpose. For primary thalamic receiving populations, we labeled functionally adjacent regions with names that were associated with these populations. Thus, for the visually-activated areas adjacent to and near the entopallium that have been called lateral nidopallium (LN) and lateral ventral mesopallium (LMV), we called them nidopallium adjacent to the entopallium (Ne) and ventral mesopallium near the entopallium (MVe). For somatosensory areas near basorostralis, we called them Nb and MVb. For the auditory areas near Field L2, we called them N-L2 (for L1 and L3), and MV-L2 (for caudal mesopallium, CM); we note here that based on the FoxP1 expression, CM and the vocal nuclei Av and MO of songbirds, the MO of parrots, and the VAM of hummingbirds are all within the ventral mesopallium. This naming scheme allowed us to easily compare expression patterns across species.

Finally, while the presence of seven comparable cerebral vocal nuclei amongst the three vocal learning bird groups has been published [5]–[7] and reviewed [10], we briefly review the evidence, particularly for the lesser studied nuclei, to help clarify the definitions used in this study. We define a vocal nucleus as a continuous anatomical structure that has vocalizing-associated activation. In this regard, HVC, RA, NIf, Av, MO, and Area X of songbirds can be defined as one structure each. LMAN and MMAN in zebra finches has been noted to be discontinuous [71], [80]. However, we noted that this is not the case for all songbirds. In canaries, for example, LMAN and MMAN are one continuous structure (**Fig. S9A**). Further, in zebra finches, in the central part of LMAN and MMAN, a bridge of singing-activated neurons connects LMAN and MMAN (**Fig. S9B**). Therefore, we consider LMAN and MMAN as one nucleus with different lateral and medial domains, as the names imply. For Av and MO in songbirds, these nuclei have been repeatedly identified in singing-driven IEG expression studies [5], [33], [125]; Av was initially identified as a nucleus that receives a projection from HVC [127]; the connectivity of songbird MO is not yet known. For budgerigars, both ZENK and c-fos have been used to identify all seven vocal nuclei [6], [128], and many connectivity studies have been performed [30], [76] (reviewed in [10]). For hummingbirds, only ZENK has been used to identify the vocal nuclei, and one connectivity study performed [79], but all seven nuclei have been found in at least five species ([7] and this study). Thus, while further work is necessary to determine the specific functions and analogies of these nuclei within and across vocal learning bird orders, the current evidence supports their presence.

## Supporting Information

Figure S1 Behavioral apparatuses used in this study. A. Cylindrical plexiglass cage with a circular perch placed in the center of the cage used to detect wing whirring and other behaviors. Infrared-sensitive cameras (IR) allowed for constant observation even under dim light conditions [26], [27]. B. Rotating plexiglass wheel used to induce hopping behavior in zebra finches and budgerigars. When the wheel was externally driven by the motor, the birds hopped in order to stay upright.
(1.02 MB TIF)

Figure S2 Anatomical definitions of brain areas and higher magnifications of posterior movement-associated areas. A. GluR1 expression in sagittal sections of a garden warbler male brain, which defines major anatomical subdivisions and some vocal nuclei. Anterior is right, dorsal is up. Scale bar, 2 mm. B. High magnification of ZENK expression in sagittal sections with garden warbler vocal nuclei HVC (a) and RA (d), indicated by black arrows, and sections laterally adjacent, showing the transition from the vocal nuclei to the movement-associated areas DLN (c) and LAI (f), indicated by white arrows, in a bird that performed flights during the day light. Dashed line in (f) shows the boundary between the arcopallium and nidopallium dorsal to it and striatum anterior to it. Scale bar, 0.5 mm.
(5.46 MB TIF)

Figure S3 Zebra finch serial coronal brain sections. A (a, a'). Right hemisphere sections of ZENK expression from a male bird that hopped in the rotating wheel in the dark while deaf. (b, b') FoxP1 expression on adjacent sections are shown to help define anatomical regions in (c, c') the corresponding anatomical profile drawings; red lines: areas with movement-induced expression. In three sections (a: rows 1, 4, and 5), we accidentally hybridized ZENK and FoxP1 simultaneously, effectively performing double-labelling; we show the results here as they further help define the boundaries of ZENK expression (higher signal intensity) with FoxP1 (lower signal intensity). Top left row: anterior-most sections; bottom right row: posterior-most. Scale bar, 2 mm. B. Higher magnification of ZENK expression in frontal sections showing (a) DLN and (b) LAI. Hopping-induced expression in DLN (a) is caudal and lateral to HVC (not shown); expression in LAI is lateral to RA in this section. The darkly stained arched curve above RA and LAI is the boundary between the arcopallium and nidopallium. Medial is left, dorsal up; right hemisphere. Scale bar, 0.5 mm.
(8.79 MB TIF)

Figure S4 High power brightfield and false-color images of posterior areas. A. HVC, PLN and DLN. B. NIf, Av, PLN and PLMV. C. RA and LAI. Purple label: Nissl cresyl violet stain; black label: silver grains from labelled ZENK mRNA in neurons. D. False-colored images of singing- and movement-associated ZENK expression in (a, green) in NIf and Av of an adult zebra finch male that sang for 30 min and was deaf [from Fig. 7B], (b, magenta) in PLN and PLMV of an adult zebra finch male that hopped for 30 min and was deaf [from panel Ab and Fig. 7C], and (c) overlap between the expression patterns of (a) and (b). The expression anterior to NIf in (a) appears to not be neither hearing, singing or hopping-associated, as it can occur whether or not the animals sing or are deaf, and it did not occur when they hopped. Anterior is right, dorsal is up. Scale bar, 200 µm.
(8.84 MB TIF)

Figure S5 Examples of widespread ZENK expression. A. Example of widespread ZENK expression in a zebra finch male that had performed many behaviours, including hopping, eating, singing to a female, and seeing light for the first time in the morning. B. Example of widespread ZENK expression in an Anna's Hummingbird male that had been performing many behaviours in the early morning (1h after sunrise), including flying, feeding, and chasing other birds. Medial is left, dorsal up. Scale bar, 2 mm.
(5.31 MB TIF)

Figure S6 Budgerigar coronal brain sections. Shown is ZENK expression in the right hemisphere. A. Vocal areas: perched singing bird while alone and moving relatively little. B. Movement areas: bird hopping in the rotating wheel in the dark while deaf. C. FoxP1 expression from adjacent sections of the bird in (B). D. Corresponding anatomical drawings; red: areas with movement-induced expression. First row are anterior-most sections. Note the absence of a distinct boundary in the ZENK expression between LAI and SLN, which can be seen with FoxP1 expression. Also note that ASt in the middle section is medial to the MMSt vocal nucleus in the same section, but caudal to MMSt as revealed by the more anterior (top row) section, consistent with the sagittal series (Fig. 10Ac). For the large area of expression between B and the LAM and LAN vocal nuclei, there is no distinct boundary. Medial is left, dorsal is up. Scale bar, 2 mm.
(9.74 MB TIF)

Figure S7 Anna's hummingbird serial coronal sections. Shown is ZENK expression in both the experimental and control hemispheres. A. Vocal and other areas: right hemisphere of a bird that was singing interspersed with flying near and feeding from an outdoor feeder in the morning. B. Auditory, visual, and movement areas: control (contralateral to open eye and ear) and experimental (contralateral to covered eye and ear) hemispheres of a bird hovering in a plexiglass cage in dim light. C. FoxP1 expression from adjacent sections of the bird in (B). D. Corresponding anatomical drawings; red: areas with movement-induced expression; blue: areas with auditory- or visual-induced expression (auditory areas also determined from a previous study [7]). First row are rostral-most sections. Note that in the singing and flying animal there is high levels of ZENK expression in vocal nuclei and many other brain areas, but in the hovering animal, most areas of highest induced expression are closest to the vocal nuclei and not different between experimental and control hemispheres. Dorsal is up. Scale bar, 2 mm.
(6.35 MB TIF)

Figure S8 Movement-induced ZENK expression in vocal non-learning female songbirds. A. Example comparison of garden warbler male and female anterior brain areas from animals that performed wing whirring movements during migratory restlessness. Females have atrophied vocal nuclei, but show movement-associated expression in areas corresponding to the location of vocal nuclei in males. Scale bar, 0.5 mm. B. Example sagittal sections showing anterior (left) and posterior (right) areas of a zebra finch female hopping in the dark while deaf. As seen in garden warblers, the female finches have atrophied vocal nuclei but similar movement-associated expression as the males. Rostral is right, dorsal is up. Scale bar, 2 mm.
(6.16 MB TIF)

Figure S9 Continuity of MMAN and LMAN in songbirds. A. Coronal section of ZENK expression of an adult male canary that sang undirected song for 30 min. MMAN and LMAN are contiguous. B. Coronal section of ZENK expression of an adult zebra finch male that sang undirected song for 30 min. There is a bridge of singing-activated neurons between the cores of MMAN and LMAN. The medial part of Area X is in more caudal sections. (A) is from a non-radioactive in-situ (Dig probe) and (B) from a radioactive in-situ (^35^S probe), both in brightfield views. Scale bar 2mm.
(2.94 MB TIF)
