# Supplementary figures and images for: Molecular Mapping of Movement-Associated Areas in the Avian Brain: A Motor Theory for Vocal Learning Origin
Source: PLoS One. 2008 Mar 12;3(3):e1768. doi: 10.1371/journal.pone.0001768 (PMC2258151; doi:10.1371/journal.pone.0001768)

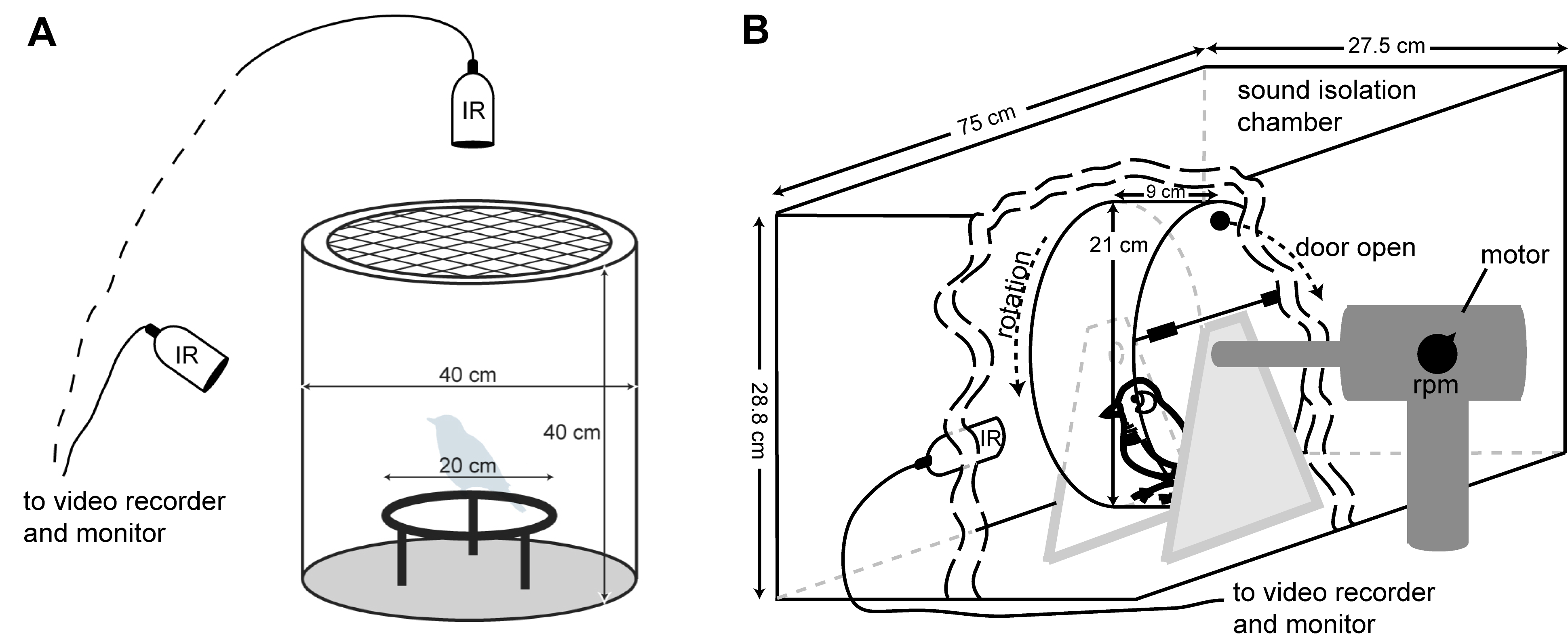

Supplement: Figure S1 — Behavioral apparatuses used in this study. A. Cylindrical plexiglass cage with a circular perch placed in the center of the cage used to detect wing whirring and other behaviors. Infrared-sensitive cameras (IR) allowed for constant observation even under dim light conditions [26], [27]. B. Rotating plexiglass wheel used to induce hopping behavior in zebra finches and budgerigars. When the wheel was externally driven by the motor, the birds hopped in order to stay upright. (1.02 MB TIF) [file pone.0001768.s001.tif]

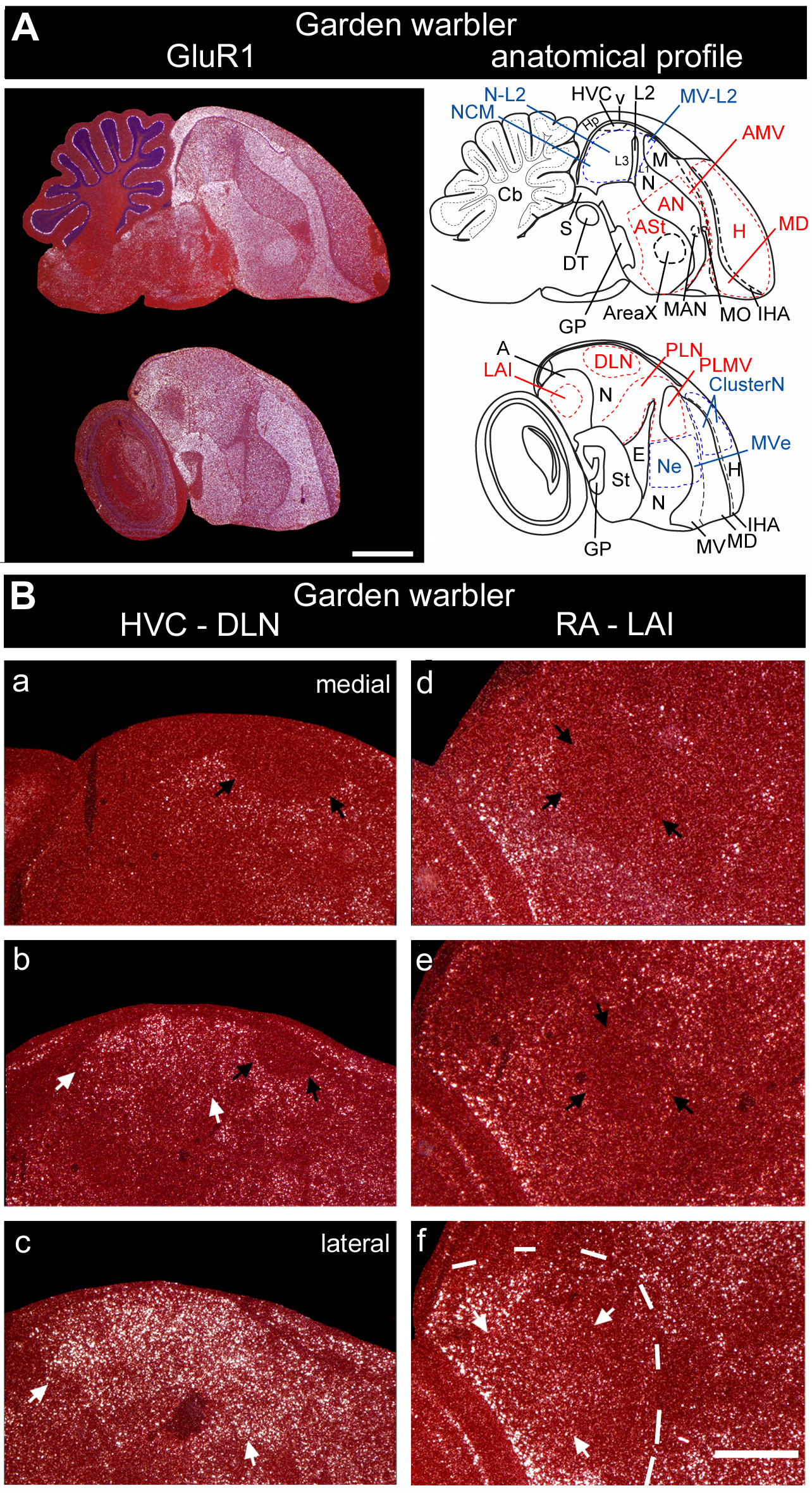

Supplement: Figure S2 — Anatomical definitions of brain areas and higher magnifications of posterior movement-associated areas. A. GluR1 expression in sagittal sections of a garden warbler male brain, which defines major anatomical subdivisions and some vocal nuclei. Anterior is right, dorsal is up. Scale bar, 2 mm. B. High magnification of ZENK expression in sagittal sections with garden warbler vocal nuclei HVC (a) and RA (d), indicated by black arrows, and sections laterally adjacent, showing the transition from the vocal nuclei to the movement-associated areas DLN (c) and LAI (f), indicated by white arrows, in a bird that performed flights during the day light. Dashed line in (f) shows the boundary between the arcopallium and nidopallium dorsal to it and striatum anterior to it. Scale bar, 0.5 mm. (5.46 MB TIF) [file pone.0001768.s002.tif]

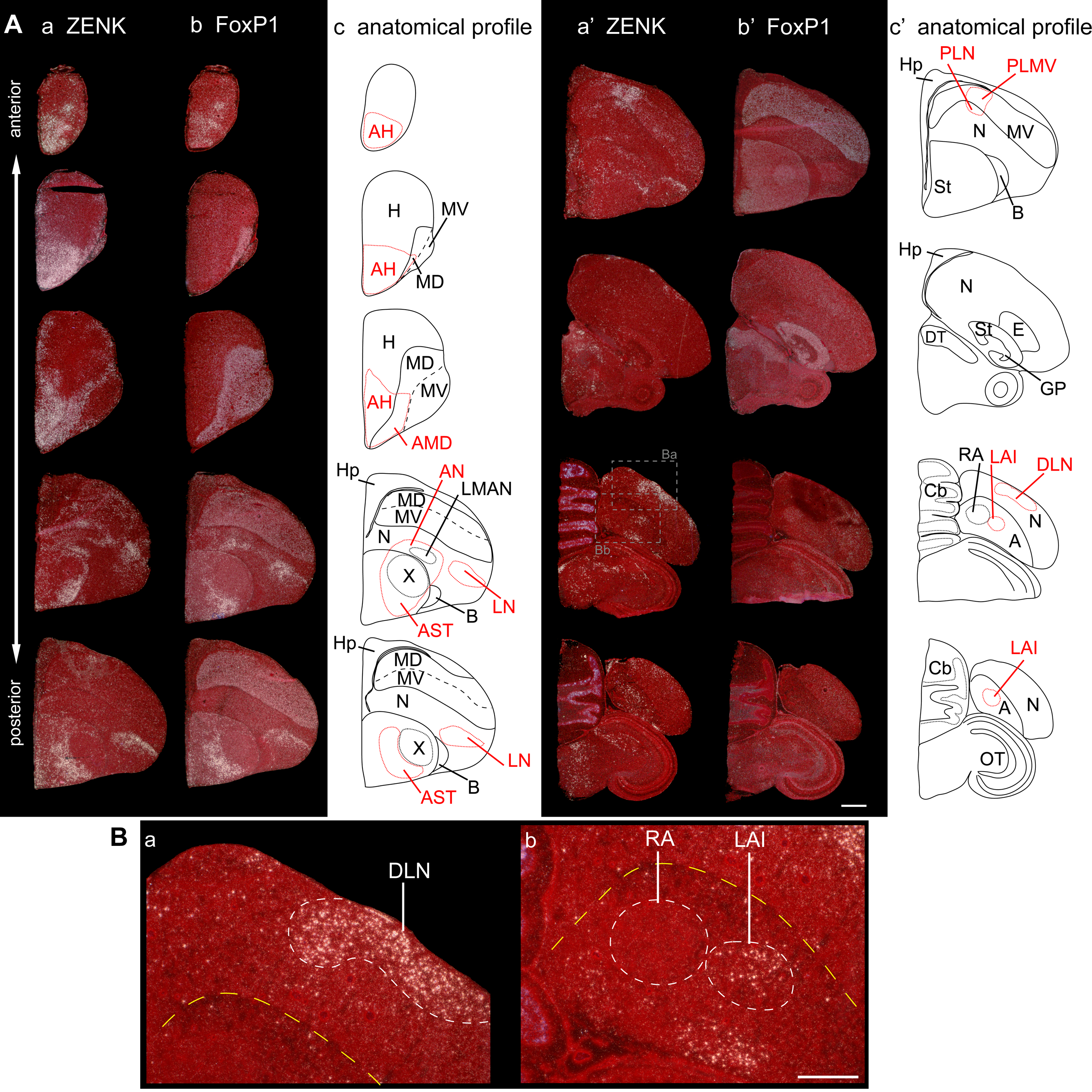

Supplement: Figure S3 — Zebra finch serial coronal brain sections. A (a, a'). Right hemisphere sections of ZENK expression from a male bird that hopped in the rotating wheel in the dark while deaf. (b, b') FoxP1 expression on adjacent sections are shown to help define anatomical regions in (c, c') the corresponding anatomical profile drawings; red lines: areas with movement-induced expression. In three sections (a: rows 1, 4, and 5), we accidentally hybridized ZENK and FoxP1 simultaneously, effectively performing double-labelling; we show the results here as they further help define the boundaries of ZENK expression (higher signal intensity) with FoxP1 (lower signal intensity). Top left row: anterior-most sections; bottom right row: posterior-most. Scale bar, 2 mm. B. Higher magnification of ZENK expression in frontal sections showing (a) DLN and (b) LAI. Hopping-induced expression in DLN (a) is caudal and lateral to HVC (not shown); expression in LAI is lateral to RA in this section. The darkly stained arched curve above RA and LAI is the boundary between the arcopallium and nidopallium. Medial is left, dorsal up; right hemisphere. Scale bar, 0.5 mm. (8.79 MB TIF) [file pone.0001768.s003.tif]

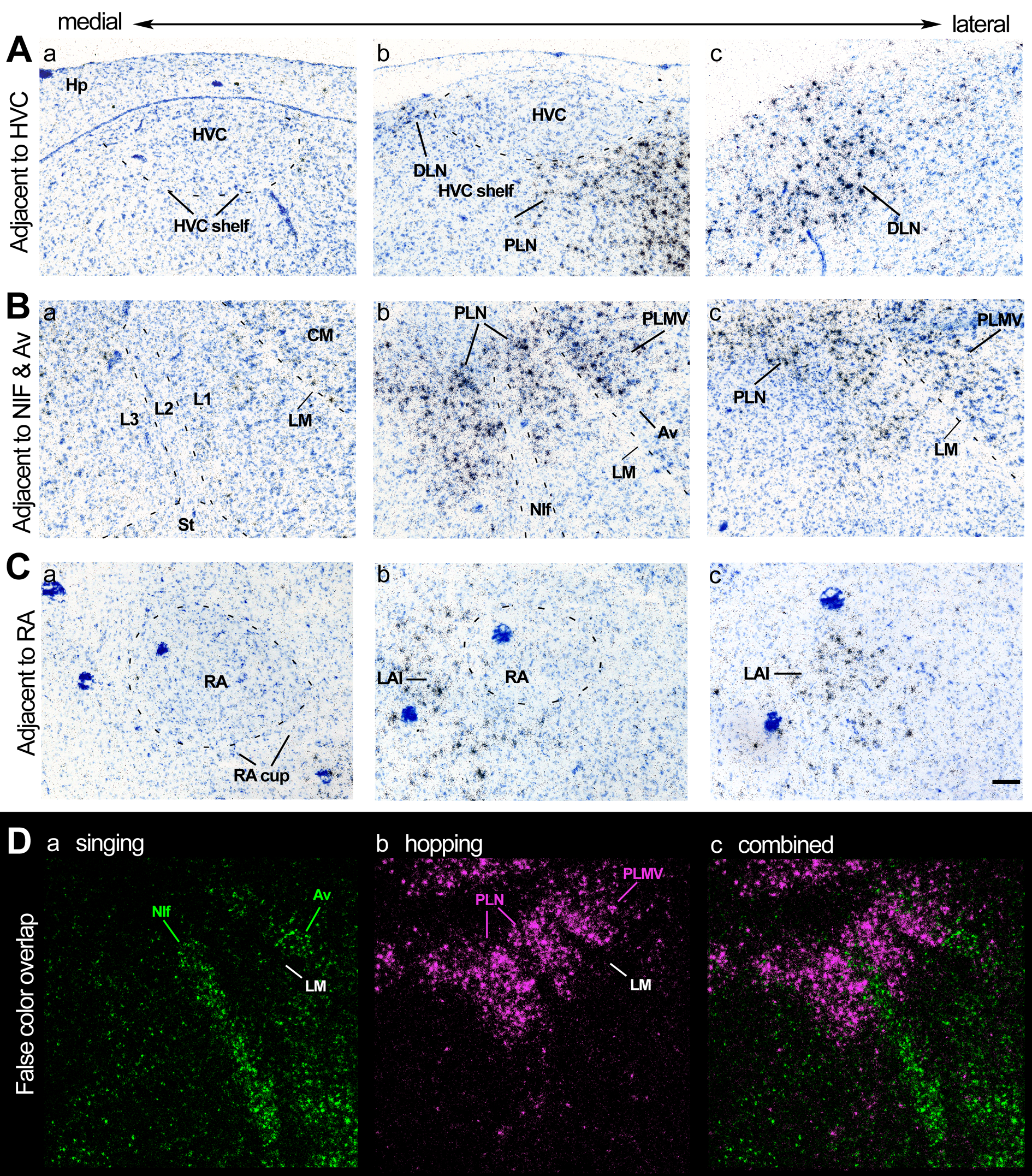

Supplement: Figure S4 — High power brightfield and false-color images of posterior areas. A. HVC, PLN and DLN. B. NIf, Av, PLN and PLMV. C. RA and LAI. Purple label: Nissl cresyl violet stain; black label: silver grains from labelled ZENK mRNA in neurons. D. False-colored images of singing- and movement-associated ZENK expression in (a, green) in NIf and Av of an adult zebra finch male that sang for 30 min and was deaf [from Fig. 7B], (b, magenta) in PLN and PLMV of an adult zebra finch male that hopped for 30 min and was deaf [from panel Ab and Fig. 7C], and (c) overlap between the expression patterns of (a) and (b). The expression anterior to NIf in (a) appears to not be neither hearing, singing or hopping-associated, as it can occur whether or not the animals sing or are deaf, and it did not occur when they hopped. Anterior is right, dorsal is up. Scale bar, 200 µm. (8.84 MB TIF) [file pone.0001768.s004.tif]

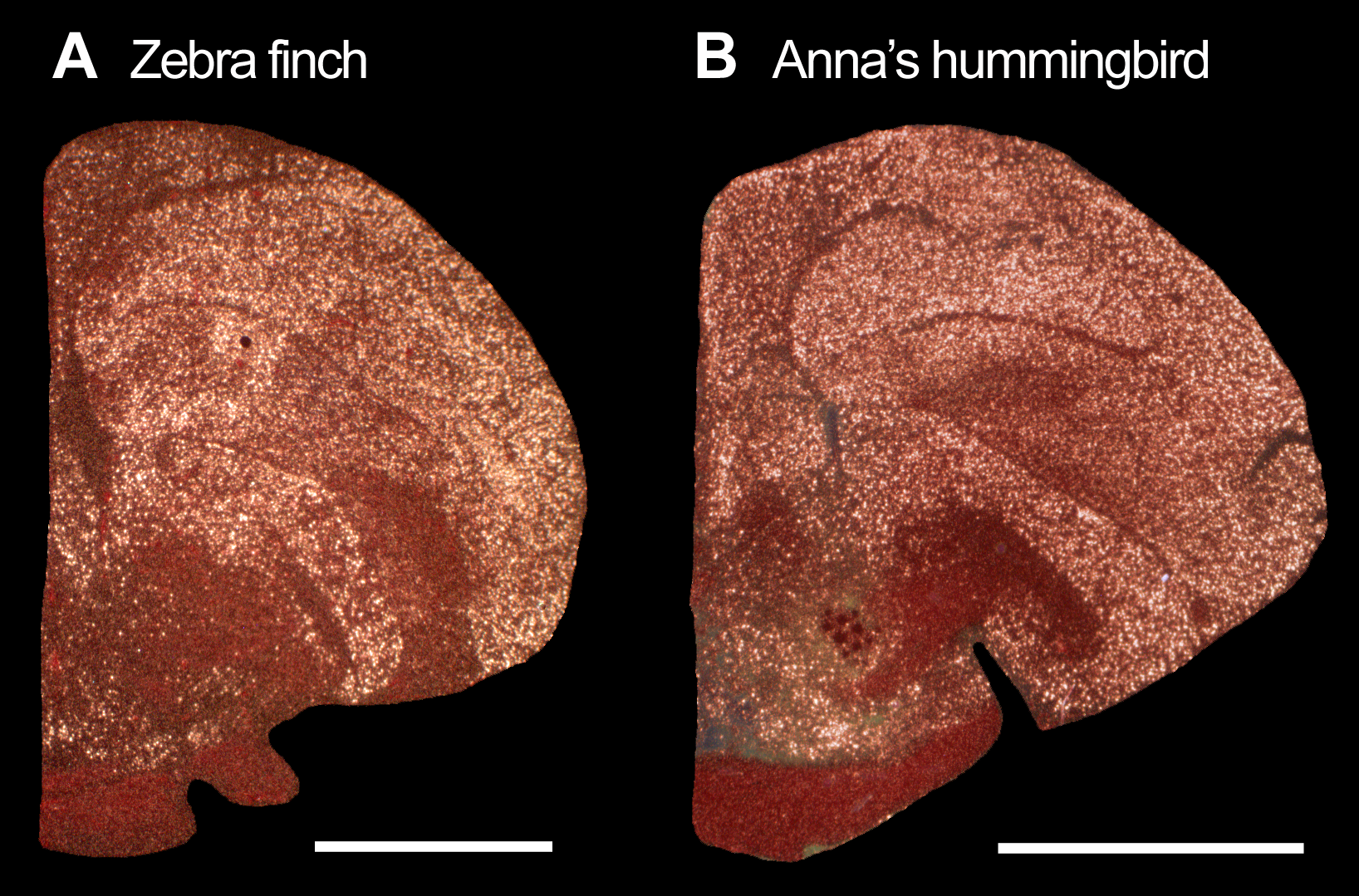

Supplement: Figure S5 — Examples of widespread ZENK expression. A. Example of widespread ZENK expression in a zebra finch male that had performed many behaviours, including hopping, eating, singing to a female, and seeing light for the first time in the morning. B. Example of widespread ZENK expression in an Anna's Hummingbird male that had been performing many behaviours in the early morning (1h after sunrise), including flying, feeding, and chasing other birds. Medial is left, dorsal up. Scale bar, 2 mm. (5.31 MB TIF) [file pone.0001768.s005.tif]

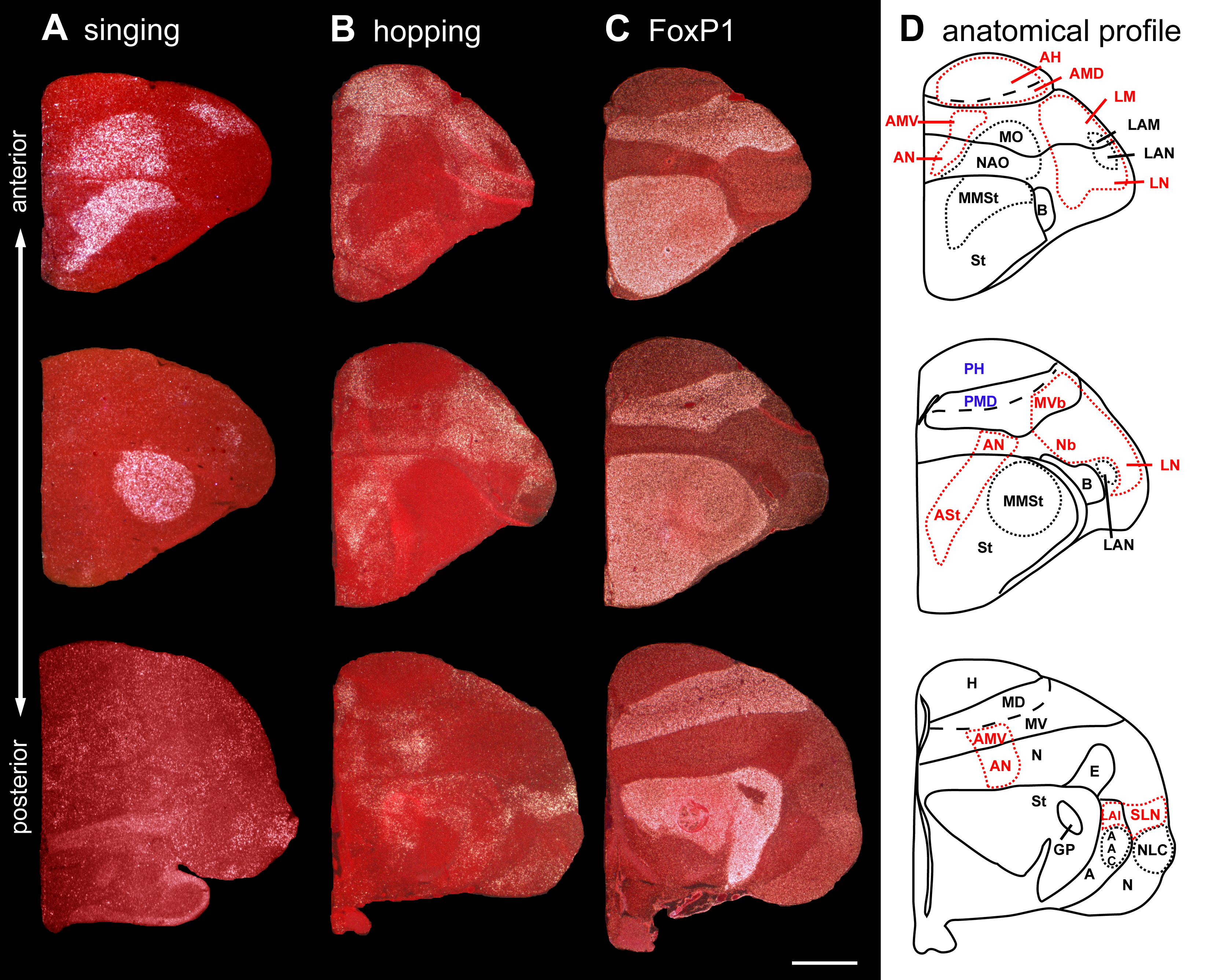

Supplement: Figure S6 — Budgerigar coronal brain sections. Shown is ZENK expression in the right hemisphere. A. Vocal areas: perched singing bird while alone and moving relatively little. B. Movement areas: bird hopping in the rotating wheel in the dark while deaf. C. FoxP1 expression from adjacent sections of the bird in (B). D. Corresponding anatomical drawings; red: areas with movement-induced expression. First row are anterior-most sections. Note the absence of a distinct boundary in the ZENK expression between LAI and SLN, which can be seen with FoxP1 expression. Also note that ASt in the middle section is medial to the MMSt vocal nucleus in the same section, but caudal to MMSt as revealed by the more anterior (top row) section, consistent with the sagittal series (Fig. 10Ac). For the large area of expression between B and the LAM and LAN vocal nuclei, there is no distinct boundary. Medial is left, dorsal is up. Scale bar, 2 mm. (9.74 MB TIF) [file pone.0001768.s006.tif]

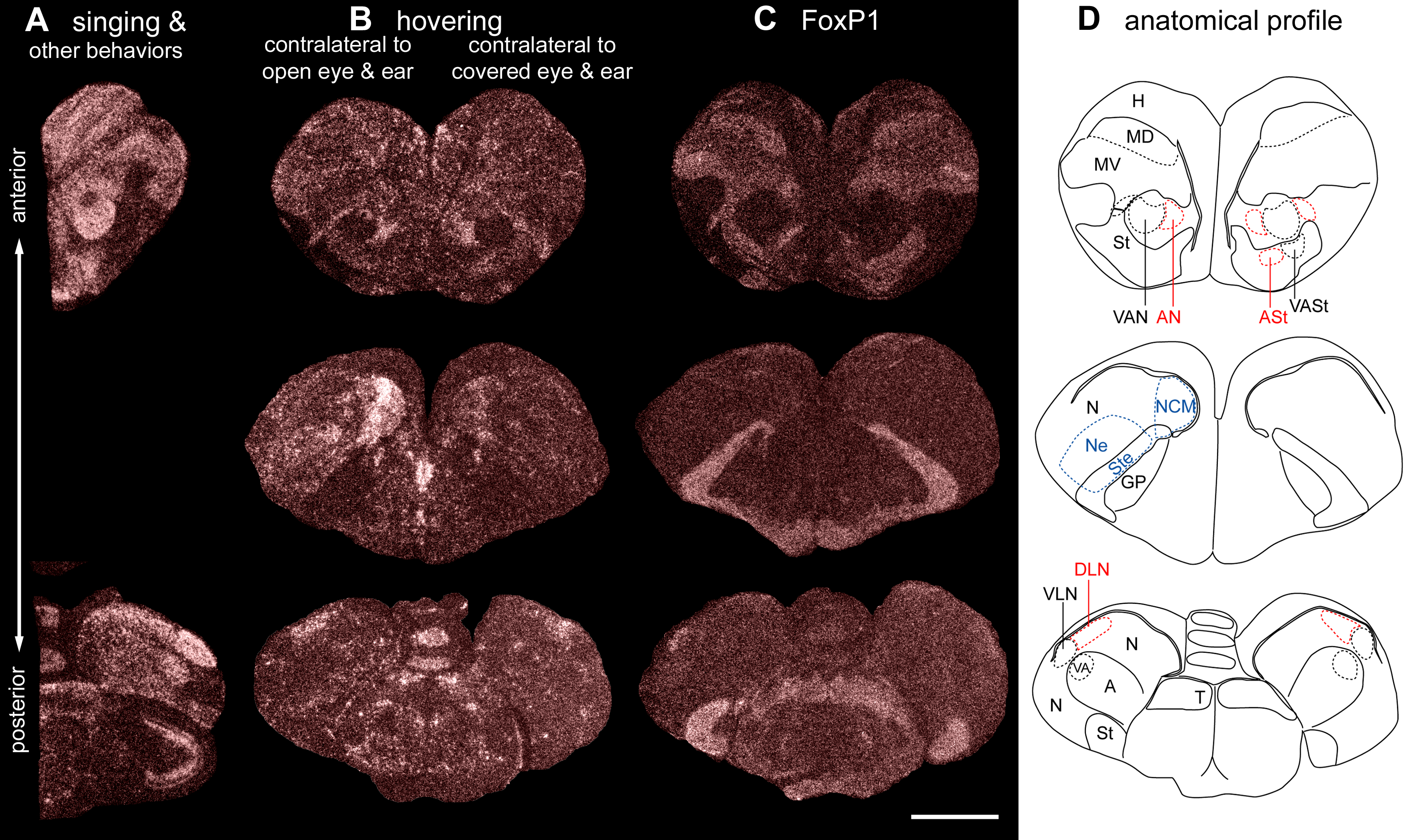

Supplement: Figure S7 — Anna's hummingbird serial coronal sections. Shown is ZENK expression in both the experimental and control hemispheres. A. Vocal and other areas: right hemisphere of a bird that was singing interspersed with flying near and feeding from an outdoor feeder in the morning. B. Auditory, visual, and movement areas: control (contralateral to open eye and ear) and experimental (contralateral to covered eye and ear) hemispheres of a bird hovering in a plexiglass cage in dim light. C. FoxP1 expression from adjacent sections of the bird in (B). D. Corresponding anatomical drawings; red: areas with movement-induced expression; blue: areas with auditory- or visual-induced expression (auditory areas also determined from a previous study [7]). First row are rostral-most sections. Note that in the singing and flying animal there is high levels of ZENK expression in vocal nuclei and many other brain areas, but in the hovering animal, most areas of highest induced expression are closest to the vocal nuclei and not different between experimental and control hemispheres. Dorsal is up. Scale bar, 2 mm. (6.35 MB TIF) [file pone.0001768.s007.tif]

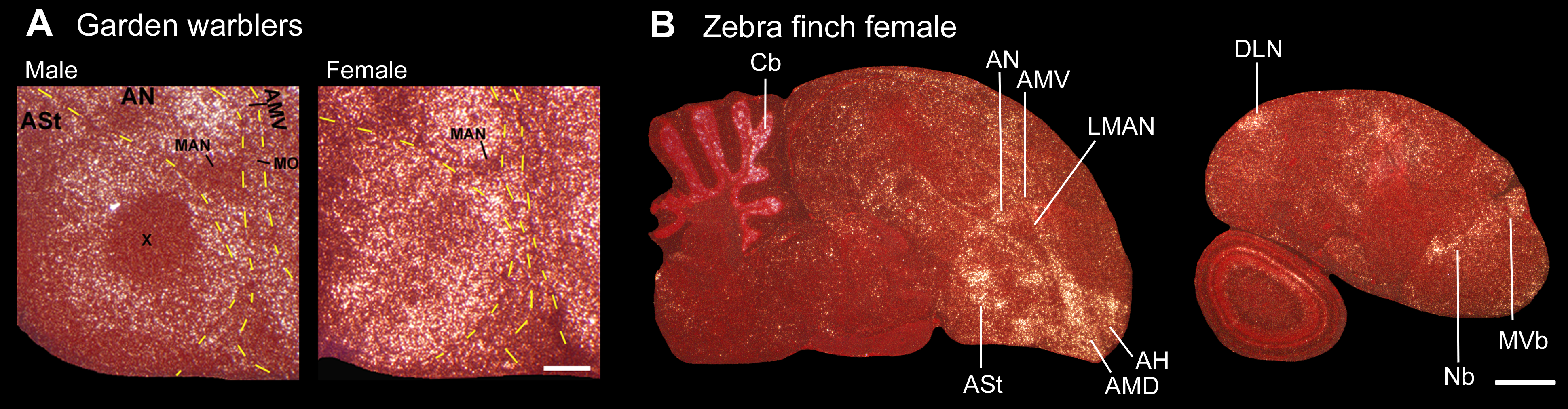

Supplement: Figure S8 — Movement-induced ZENK expression in vocal non-learning female songbirds. A. Example comparison of garden warbler male and female anterior brain areas from animals that performed wing whirring movements during migratory restlessness. Females have atrophied vocal nuclei, but show movement-associated expression in areas corresponding to the location of vocal nuclei in males. Scale bar, 0.5 mm. B. Example sagittal sections showing anterior (left) and posterior (right) areas of a zebra finch female hopping in the dark while deaf. As seen in garden warblers, the female finches have atrophied vocal nuclei but similar movement-associated expression as the males. Rostral is right, dorsal is up. Scale bar, 2 mm. (6.16 MB TIF) [file pone.0001768.s008.tif]

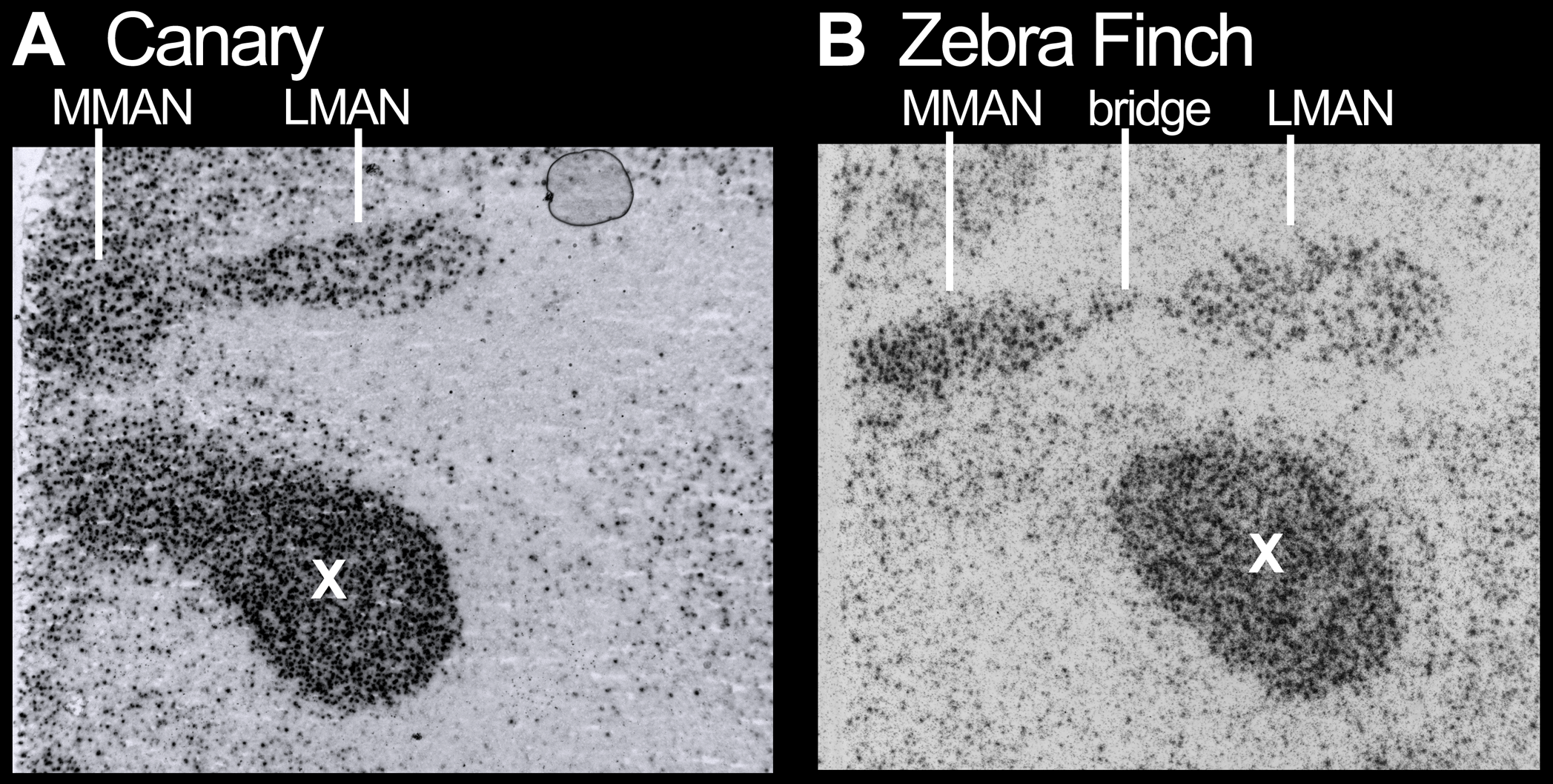

Supplement: Figure S9 — Continuity of MMAN and LMAN in songbirds. A. Coronal section of ZENK expression of an adult male canary that sang undirected song for 30 min. MMAN and LMAN are contiguous. B. Coronal section of ZENK expression of an adult zebra finch male that sang undirected song for 30 min. There is a bridge of singing-activated neurons between the cores of MMAN and LMAN. The medial part of Area X is in more caudal sections. (A) is from a non-radioactive in-situ (Dig probe) and (B) from a radioactive in-situ (35S probe), both in brightfield views. Scale bar 2mm. (2.94 MB TIF) [file pone.0001768.s009.tif]
